# Ni-Catalyzed Homoallylation of Polyhydroxy *N*,*O*-Acetals with Conjugated Dienes Promoted by Triethylborane

**DOI:** 10.3390/molecules19079288

**Published:** 2014-07-02

**Authors:** Takamichi Mori, Yusuke Akioka, Gen Onodera, Masanari Kimura

**Affiliations:** Graduate School of Engineering, Nagasaki University, 1-14, Bunkyo machi, Nagasaki 852-8521, Japan

**Keywords:** nickel, homoallylation, *N*,*O-*acetal, carbohydrate, conjugated diene, triethylborane, reductive coupling

## Abstract

In the presence of Ni-catalyst and triethylborane, *N*,*O*-acetals prepared from glycolaldehyde and glyceraldehyde with primary amines *in situ* underwent homoallylation with conjugated dienes to provide 2-amino-5-hexenols in high regio- and stereoselectivity. Under similar reaction conditions, *N*,*O*-acetals from carbohydrates with primary amines provided the corresponding polyhydroxy-bishomoallylamines in good to reasonable yields.

## 1. Introduction

Ni-catalyzed C-C bond formation is a useful strategy for organic syntheses [1]. Cross-coupling of organometallic compounds with aromatic halides, as well as allylation and vinylation of carbonyls, is widely utilized for the synthesis of physiologically active molecules and fine chemicals [2]. Compared to catalytically C-C bond transformations involving allylation and vinylation, homoallylation of carbonyls providing bis-homoallyl alcohols have serious limitations, which may be due to the unavailability and low stability of homoallyl anion species that can react with electrophiles [3,4].

Recently, a Ni catalyst was developed that could promote the homoallylation of benzaldehyde with a wide variety of 1,3-dienes in the presence of triethylborane to afford bis-homoallyl alcohols (Equation (1)) [5,6]. For these processes, isoprene reacts at the C1 position with an aromatic aldehyde to give 3-methyl-4-penten-1-ol with excellent 1,3-*anti* stereoselectivity. A similar homoallylation to produce aliphatic aldehydes and ketones was successful using diethylzinc instead of triethylborane [7]. Results indicated that diethylzinc functions as a more effective promoter than triethylborane for the homoallylation of aliphatic aldehydes and ketones. In contrast, triethylborane is compatible with water and alcohols, and even promotes homoallylation of aqueous aldehyde (e.g., glutaraldehyde) and ω-hydroxyaldehyde (lactol) with conjugated dienes to afford ω-hydroxyhomoallyl alcohols (Equation (2)) [8]. Thus, triethylborane and diethylzinc can be used in a complementary manner to accelerate homoallyation of carbonyl compounds.

In addition, Ni-catalyzed homoallylation of aldimines prepared from aldehydes and primary amines *in situ* with conjugated dienes provided bis-homoallylamines in high regio- and stereoselectivity (Equation (3)) [9]. Thus, the C1 position of isoprene reacts with aldimines to afford 3-methyl-4-pentenylamines with excellent 1,3-*syn* stereoselectivity, compared to 1,3-*anti* stereoselectivity when using aldehydes.

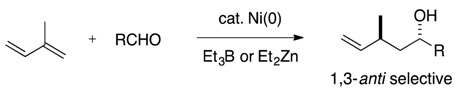
(1)

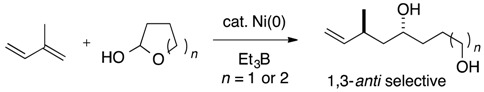
(2)

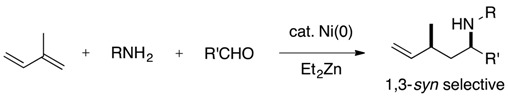
(3)


This report describes a similar reaction system involving a Ni catalyst and triethylborane that was extended successfully to the homoallylation of *N*,*O*-acetals prepared from cyclic hemiacetals and primary amines to provide ω-hydroxybishomoallylamines in high regio- and moderate stereoselectivity (Equation (4)). In similar catalytic reaction systems, *N*,*O*-acetals from carbohydrates with primary amines gave the polyhydroxybishomoallylamines in good to reasonable yields.

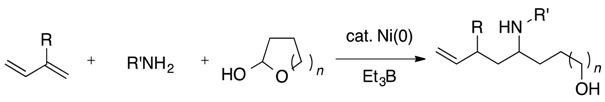
(4)


## 2. Results and Discussion

Results of reactions of isoprene with *N*,*O*-acetals prepared from cyclic hemiacetals and *p*-methoxyaniline are summarized in Table 1 (Equation (5), see, experimental section and supporting information). Reactions were conducted at room temperature using isoprene, Ni(cod)_2_ catalyst, triethylborane, and *N*,*O*-acetals under nitrogen atmosphere. Isoprene reacted at the C1 position with *N*,*O*-acetals and underwent homoallylation to provide hydroxybishomoallylamines. 2-Hydroxytetrahydrofuran provided 1-(3-hydroxypropyl)-3-methyl-4-pentenylamine **1a** in reasonable yield along with a mixture of diastereomers in a 5:1 ratio (Table 1, entry 1). 5-Naphthyl-2-hydroxytetrahydrofuran provided the desired product **1b** in 71% yield along with two diastereomers in a 2:1 ratio (Table 1, entry 2). 5-Methyl-5*-n*-hexyl-2-hydroxytetrahydrofuran participated in the homoallylation to afford hydroxylamine **1c**, which possessed a tertiary alcohol moiety (Table 1, entry 3). Six-membered cyclic hemiacetals could be used for homoallylation to form amino alcohols **1d** and **1e** in 6:1 and 4:1 ratios, respectively (Table 1, entries 4 and 5). 2-Hydroxychroman served as an *N*,*O*-acetal precursor by treatment with a primary amine to provide *o-*aminoalkyl phenol **1f** (Table 1, entry 6). *N*-Boc-2-hydroxypiperidine acted as an aldimine in the presence of *p*-methoxyaniline to participate in the coupling reaction with isoprene to provide 2-butenylaminobishomoallylamine **1g** (Table 1, entry 7). Seven-membered cyclic hemiacetal underwent a similar homoallylation to provide 1-(5-hydroxypentyl)-3-methyl-4-pentenylamine **1h** in reasonable yield (Table 1, entry 8).

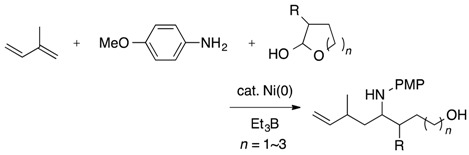
(5)


**Table 1:** Ni-Catalyzed homoallylation of *N*,*O*-acetals with isoprene *^a^*.

Entry	Hemiacetal	Product	Yield (%) [Ratio]
1	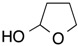	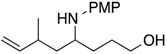	**1a**: 58% [5:1]
2	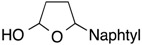	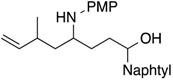	**1b**: 71% [2:1]
3	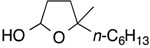	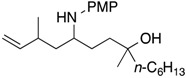	**1c**: 59% [3:1]
4	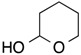	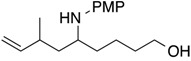	**1d**: 91% [6:1]
5	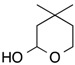	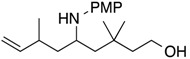	**1e**: 69% [4:1]
6	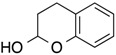	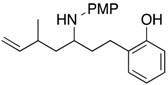	**1f**: 66% [7:1]
7	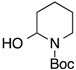	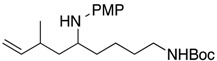	**1g**: 41% [3:1]
8	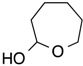	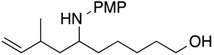	**1h**: 58% [4:1]

*^a^* *N*,*O*-Acetals were prepared from cyclic hemiacetals (1 mmol) and amines (2 mmol) stirring in THF (2 mmol). A solution of isoprene (4 mmol), Ni(cod)_2_ (0.1 mmol) in THF (2 mL) and Et_3_B (3.6 mmol) were introduced to the *N*,*O*-acetals, and the reaction mixture was stirred at room temperature for 24 h under N_2_.

Glycolaldehyde dimer is a two-carbon monosaccharide (diose) that is an important component of biologically active molecules [10,11]. The reaction of glycolaldehyde dimer as an *N*,*O*-acetal precursor with conjugated dienes to furnish 1-hydroxymethyl-4-pentenylamines also was examined. Results using various conjugated dienes and *N*,*O*-acetals prepared from primary amines and glycolaldehyde dimer are summarized in Table 2 (Equation (6)). 1,3-Butadiene reacted with *N*,*O*-acetal prepared from *p*-methoxyaniline to yield 47% of 1-hydroxymethyl-4-pentenylamine **1i** along with 23% of the internal olefin isomer **1i’** (Table 2, entry 1). *N*,*O*-Acetal from aniline underwent homoallylation with isoprene to provide 1-hydroxymethyl-3-methyl-4-pentenylamine **1j** in reasonable yield with a diastereomeric mixture in a 8:1 ratio (Table 2, entry 2). *o*-Methoxy and *p*-methoxyaniline participated in similar homoallylations to provide hydroxyamines **1k** and **1l**, respectively, with high stereoselectivity in an 8:1 ratio and as a single isomer, respectively (Table 2, entries 3 and 4). *p*-Bromoaniline underwent homoallylation effectively to afford the desired hydroxylamine **1m** as the sole product (Table 2, entry 5). Benzylamine yielded an intractable mixture; the expected reaction was not observed (Table 2, entry 6). Myrcene also participated in homoallylation with *N*,*O*-acetal to produce the corresponding hydroxylamine **1n** as a single product (Table 2, entry 7).

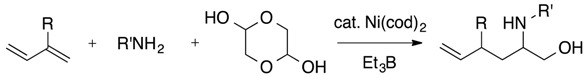
(6)


**Table 2:** Homoallylation of *N*,*O*-acetals prepared from glycolaldehyde dimer *^a^*.

Entry	Diene: R	Amine: R’	Product, Yield (%) [Ratio]
1	H	*p*-methoxyphenyl	**1i**: 47% *^b^*
2	Me	phenyl	**1j**: 64% [8:1]
3	Me	*o*-methoxyphenyl	**1k**: 64% [8:1]
4	Me	*p*-methoxyphenyl	**1l**: 59% [single]
5	Me	*p*-bromophenyl	**1m**: 50% [single]
6	Me	benzyl	intractable mixture
7	-(CH_2_)_2_CH=CMe_2_	*p*-methoxyphenyl	**1n**: 49% [single]

*^a^* *N*,*O*-Acetals were prepared from glycolaldehyde dimer (1 mmol) and amines (4 mmol) stirring in THF (2 mmol). A solution of conjugated diene (8 mmol), Ni(cod)_2_ (0.1 mmol) in THF (2 mL) and Et_3_B (6 mmol) were introduced to the *N*,*O*-acetals, and the reaction mixture was stirred at room temperature for 48 h under N_2_. *^b^* Internal olefin isomer **1i’** was obtained in 23%.

Glyceraldehyde is a triose monosaccharide generally used for the enzymatic synthesis of d-fructose and l-sorbose with aldolases [12]. The glyceraldehyde can serve as an important electrophilic component for coupling reactions to form a physiologically active molecules. The Ni-catalyzed homoallylation of glyceraldehyde dimer was accomplished using primary amines and conjugated dienes in the presence of triethylborane (Table 3, Equation (7)). In this reaction, *N*,*O*-acetals prepared from glyceraldehyde dimer and primary amines in DMF via azeotropic distillation underwent homoallylation with conjugated dienes to furnish dihydroxybishomoallylamines. 1,3-Butadiene reacted with *N*,*O*-acetal from *p*-methoxyaniline to provide 1-(1,2-dihydroxyethyl)-4-pentenylamine **1o** in 56% yield along with the internal olefin isomer **1o’** in 17% yield (Table 3, entry 1). *N*,*O*-Acetal from aniline participated in homoallylation with isoprene to provide 1-(1,2-dihydroxyethyl)-3-methyl-4-pentenylamine **1p** in 62% yield in a 2:1 ratio of diastereomers (Table 3, entry 2). *p*-Methoxyaniline gave a similar homoallylation product **1q** with a mixture of diastereoisomers in a 2:1 ratio (Table 3, entry 3). Benzylamine provided an intractable mixture in the same way as the result of glycolaldehyde with aliphatic amine (Table 3, entry 4). Myrcene underwent homoallylation with *N*,*O*-acetal to produce the corresponding hydroxylamine **1r** in reasonable yield with diastereomers in a 2:1 ratio, as well as isoprene (Table 3, entry 5).

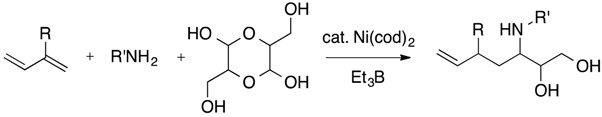
(7)


**Table 3:** Ni-Catalyzed homoallylation of *N*,*O*-acetals prepared from glyceraldehyde dimer *^a^*.

Entry	Diene: R	Amine: R’	Product, Yield (%) [Ratio]
1	H	*p*-methoxyphenyl	**1o**: 56% [3:1] *^b^*
2	Me	phenyl	**1p**: 62% [2:1]
3	Me	*p*-methoxyphenyl	**1q**: 69% [2:1]
4	Me	benzyl	intractable mixture
5	-(CH_2_)_2_CH=CMe_2_	*p*-methoxyphenyl	**1r**: 64% [2:1]

*^a^* *N*,*O*-Acetals were prepared from glyceraldehyde dimer (1 mmol) and amines (4 mmol) in DMF (2 mmol) via azeotropic distillation. A solution of conjugated diene (8 mmol), Ni(cod)_2_ (0.1 mmol) in THF (2 mL) and Et_3_B (6 mmol) were introduced to the residual oil of *N*,*O*-acetals, and then the reaction mixture was stirred at room temperature for 48 h under N_2_. *^b^* Internal olefin isomer **1o’** was obtained in 17% with 3:1 diastereoisomeric ratio.

Next, homoallylation of *N*,*O*-acetals from carbohydrates, such as 2-deoxy-d-ribose, d-ribose, and 2-deoxy-d-glucose, was investigated. Various *N*,*O*-acetals prepared from 2-deoxy-d-ribose and aromatic amines underwent homoallylation with conjugated dienes in one pot to provide polyhydroxyamines (Table 4, Equation (8)). 1,3-Butadiene reacted with *N*,*O*-acetal from 2-deoxy-d-ribose and *p*-methoxyaniline to afford the homoallylation product **1s** in moderate yield along with the allylation product as a diastereoisomeric mixture of internal olefin isomers **1s’** (Table 4, entry 1). Isoprene reacted with high regioselectivity at the C1 position with *N*,*O*-acetals derived from 2-deoxy-d-ribose and various aromatic amines to provide the desired polyhydroxyamines **1t**–**1x** as mixtures of two diastereomers in a nearly 2:1 ratio (Table 4, entries 2–6). Myrcene also participated in homoallylation as a conjugated diene and afforded the desired product **1y** in 56% in a 1:1 ratio (Table 4, entry 7). These consecutive homoallylations of *N*,*O*-acetals from 2-deoxy-d-ribose and primary amines provided bishomoallylamines as two diastereomers in 1:1 to 2:1 ratios, in spite of possible formation of four diastereoisomers. Since d-ribose and 2-deoxy-d-glucose are insoluble in THF, a series of *N*,*O*-acetals with d-ribose and 2-deoxy-d-glucose were prepared from amines in DMF via azeotropic distillation, and were used for homoallylation with isoprene to produce the expected polyhydroxyamines **2** and **3**, respectively (Equations (9) and (10)).

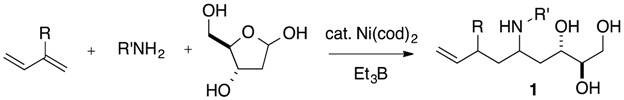
(8)


**Table 4:** Homoallylation of *N*,*O*-acetals prepared from 2-deoxy-d-ribose and amine *^a^*.

Entry	Conjugated Diene: R	Amine: R’	Product, Yield (%) [Ratio]
1	H	*p*-methoxyphenyl	**1s**: 27% [1:1] *^b^*
2	Me	*p*-methoxyphenyl	**1t**: 74% [2:1]
3	Me	*o*-methoxyphenyl	**1u**: 80% [2:1]
4	Me	3,4-dimethoxyphenyl	**1v**: 58% [2:1]
5	Me	phenyl	**1w**: 75% [2:1]
6	Me	*p*-chlorophenyl	**1x**: 30% [2:1]
7	-(CH_2_)_2_CH=CMe_2_	*p*-methoxyphenyl	**1y**: 56% [1:1]

*^a^* *N*,*O*-Acetals were prepared from 2-deoxy-d-ribose (1 mmol) and amines (2 mmol) in THF (5 mmol) via azeotropic distillation. A solution of conjugated diene (8 mmol), Ni(cod)_2_ (0.1 mmol) in THF (2 mL) and Et_3_B (6 mmol) were introduced to the *N*,*O*-acetals, and the reaction mixture was stirred at room temperature for 48 h under N_2_. *^b^* Internal olefin isomer **1s’** was obtained in 32% with 3:1 diastereoisomeric ratio.



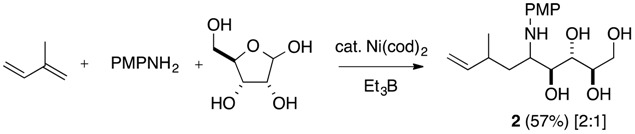
(9)

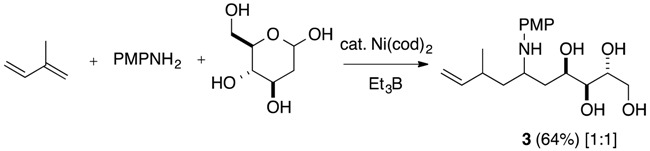
(10)


**Scheme 1:**
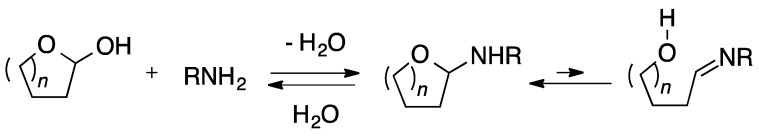
Equilibrium between cyclic *N*,*O*-acetal and ω-hydroxyamine.

Although all product absolute configurations have not been determined yet, a plausible reaction mechanism might be assumed based on the results of the homoallylation of aldimines prepared from aldehydes and primary amines with conjugated dienes [9]. *N*,*O*-Acetals were readily prepared from cyclic hemiacetals with primary amines *in situ*, and the low concentration of ω-hydroxyimine tautomers in equilibrium with the *N*,*O*-acetals appeared to promote reaction with conjugated dienes (Scheme 1). As triethylborane coordinates to the nitrogen atom of *N*,*O*-acetals as a Lewis acid, the formation of ω-hydroxyimine tautomers might predominate over *N*,*O*-acetals. An azanickelacycle intermediate *via* oxidative cyclization of conjugated dienes and ω-hydroxyimine with Ni(0) catalyst followed by σ-bond metathesis with triethylborane would result in the formation of ω-hydrox-bishomoallylamines.

## 3. Experimental

### 3.1. General

Distillation were carried out in a Kugelrohr apparatus (GTO-350RG glass tube oven, SIBATA, Soka (Saitama, Japan). Boiling points are meant to refer to the oven temperature (±1 °C). Microanalyses were performed by the Instrumental Analysis Center of Nagasaki University. Analysis agreed with the calculated values within ±0.4%. High resolution mass spectra (HRMS) were measured with a JEOL JMSDX303 instrument (JEOL, Akishima (Tokyo), Japan). Infrared spectra were recorded with a JASCO A-100 (Hachioji, Tokyo, Japan) or Shimadzu FTIR-8700 (Kyoto, Japan) infrared spectrophotometer. ^1^H (400 MHz) and ^13^C-NMR spectra (100 MHz) were measured on JEOL-GX400 instrument with tetramethylsilane as an internal standard. Chemical shift values were given in ppm downfield from the internal standard.

Tetrahydrofuran, toluene, and diethyl ether were dried and distilled from benzophenone-sodium immediately prior to use under nitrogen atmosphere. DMF was distilled over calcium chloride. Triethylborane (1 M THF, KANTO Kagaku, Tokyo, Japan), Ni(cod)_2_ (KANTO Kagaku, Tokyo, Japan) were used without further purification. Isoprene, myrcene, glycolaldehyde dimmer, glyceraldehyde dimer, 2-deoxy-d-ribose, d-ribose, 2-deoxy-d-glucose, aniline, *p*-methoxyaniline, *o*-methoxyaniline, *p*-bromoaniline, benzylamine were purchased and used without purification. 1,3-Butadiene was purchased (Tokyo Kasei Kogyo Co., Ltd., Tokyo, Japan), and was liquefied by cooling at −78 °C (dry ice/isopropanol) prior to use under argon atmosphere. 1,3-Butadiene could be measured by syringe kept cool in the freezer well beforehand, and then was introduced into the reaction mixture at room temperature. Tetrahydrofuran-2-ol, tetrahydro-2*H*-pyran-2-ol, oxepane-2-ol, 5-(naphthalen-2-yl)tetrahydrofuran-2-ol, and all of the substrates in Table 1 were prepared according to the literature [13,14,15,16].

### 3.2. Typical Procedure for Ni-catalyzed Homoallylation of N,O-acetal with Isoprene (Entry 4, Table 1)

A solution of tetrahydro-2*H*-pyran-2-ol (102 mg, 1 mmol) and *p*-anisidine (246 mg, 2 mmol) in dry THF (2 mL) was stirred overnight under nitrogen. A mixture of Ni(cod)_2_ (27.5 mg, 0.1 mmol) and isoprene (400 μL, 4 mmol) dissolved in THF (2 mL) and triethylborane (3.6 mmol, 1.0 M THF solution) were successively added to the *N*,*O*-acetal solution. The reaction mixture was stirred at room temperature for 24 h. The reaction mixture was diluted with 30 mL of EtOAc and washed with sat. NaHCO_3_, and brine. The organic phase was dried (MgSO_4_) and concentrated *in vacuo* to give a colorless oil, which was subjected to column chromatography over silica gel (hexane/EtOAc = 2/1 v/v) to give **1d** (258 mg, 91%) in a 6:1 ratio. R_f_ = 0.30 (hexane/EtOAc = 4/1 v/v).

*(4S,6S)-4-(4-Methoxyphenylamino)-6-methyloct-7-en-1-ol* (**1a**). (a mixture of major and minor isomers in a ratio of 5:1): IR (neat) 3310 (s), 3071 (s), 2924 (s), 1643 (m), 1458 (s), 1065 (s), 910 (s), 741 (s) cm^−1^; ^1^H-NMR (CDCl_3_, major-isomer) δ 0.99 (d, *J* = 6.7 Hz, 3 H), 1.60–1.69 (m, 6 H), 2.35 (qm, *J* = 6.7 Hz, 1 H), 2.52 (br, 1H), 3.32 (m, 1 H), 3.60 (t, *J* = 6.1 Hz, 2 H), 3.73 (s, 3 H), 4.92 (dm, *J* = 10.7 Hz, 1 H), 4.93 (dd, *J* = 16.9, 1.0 Hz, 1 H), 5.65 (ddd, *J* = 16.9, 10.7, 8.0 Hz, 1 H), 6.54 (dd, *J* = 6.6, 2.2 Hz, 2 H), 6.75 (m, 2 H); ^13^C-NMR (CDCl_3_, major-isomer) δ 21.0, 29.3, 32.0, 35.0, 42.5, 54.0, 55.8, 62.9, 63.0, 113.2, 114.9, 115.0, 115.4, 141.5, 141.8, 144.3; ^1^H-NMR (CDCl_3_, minor-isomer) δ 0.99 (d, *J* = 6.7 Hz, 3 H), 1.60–1.69 (m, 6 H), 2.35 (qm, *J* = 6.7 Hz, 1 H), 2.52 (br, 1H), 3.32 (m, 1 H), 3.60 (t, *J* = 6.1 Hz, 2 H), 3.73 (s, 3 H), 4.93 (dd, *J* = 16.9, 1.0 Hz, 1 H), 4.98 (dm, *J* = 17.1 Hz, 1 H), 5.65 (ddd, *J* = 16.9, 10.7, 8.0 Hz, 1 H), 6.54 (dd, *J* = 6.6, 2.2 Hz, 2 H), 6.75 (m, 2 H); ^13^C-NMR (CDCl_3_, minor-isomer) δ 20.8, 29.2, 32.0, 35.0, 45.3, 54.0, 55.8, 62.9, 63.0, 113.2, 114.8, 115.0, 115.4, 141.6, 141.8, 144.1; HRMS, calcd for C_16_H_25_NO_2_: 263.1885. Found *m/z* (relative intensity): 264.1882 (M^+^+1, 18), 263.1850 (M^+^, 97), 205.1411 (15), 204.1394 (100).

*(4S,6S)-4-(4-Methoxyphenylamino)-6-methyl-1-(naphthalenyl) octen-7-ol* (**1b**). (a mixture of major and minor isomers in a ratio of 2:1): IR (neat) 3366 (s), 2930 (s), 2359 (m), 1506 (s), 1238 (s), 1040 (s), 820 (s), 750 (s) cm^−1^; ^1^H-NMR (CDCl_3_, major-isomer) δ 0.95 (d, *J* = 6.8 Hz, 3 H), 1.38–1.94 (m, 6 H), 2.29 (qm, *J* = 6.8 Hz, 1 H), 3.11 (br, 1 H), 3.32 (br q, *J* = 5.9 Hz, 1 H), 3.71 (s, 3 H), 4.78–4.82 (m, 1 H), 4.88 (dd, *J* = 18.3, 1.9 Hz, 1 H), 4.89 (dd, *J* = 10.8, 1.9 Hz, 1 H), 5.59 (ddd, *J* = 18.3, 10.8, 8.3 Hz, 1 H), 6.53 (d, *J* = 8.9 Hz, 2 H), 6.70 (dd, *J* = 8.9, 1.0 Hz, 2 H), 7.39–7.82 (m, 7 H); ^13^C-NMR (CDCl_3_, major-isomer) δ 20.9, 31.7, 35.0, 35.5, 42.3, 53.0, 55.7, 74.5, 113.2, 114.8, 115.3, 123.9, 124.4, 125.6, 125.7, 125.9, 126.0, 127.5, 128.0, 132.8, 133.1, 141.9, 144.1, 152.2; ^1^H-NMR (CDCl_3_, minor-isomer) δ 0.92 (d, *J* = 6.8 Hz, 3 H), 1.38–1.94 (m, 6 H), 2.29 (qm, *J* = 6.8 Hz, 1 H), 3.11 (br, 1 H), 3.32 (br q, *J* = 5.9 Hz, 1 H), 3.72 (s, 3 H), 4.83 (dm, *J* = 18.3 Hz, 1 H), 4.88 (dd, *J* = 18.3, 1.9 Hz, 1 H), 4.89 (dd, *J* = 10.8, 1.9 Hz, 1 H), 5.58–5.66 (m, 1 H), 6.53 (d, *J* = 8.9 Hz, 2 H), 6.70 (dd, *J* = 8.9, 1.0 Hz, 2 H), 7.39–7.82 (m, 7 H); ^13^C-NMR (minor-isomer) δ 20.9, 31.2, 34.9, 35.7, 42.3, 52.7, 55.7, 74.4, 113.2, 114.8, 115.2, 124.0, 124.4, 125.6, 125.7, 125.9, 126.0, 127.5, 128.1, 132.6, 133.1, 141.3, 144.1, 152.1; HRMS, calcd for C_26_H_31_NO_2_: 389.2355. Found *m/z* (relative intensity): 389.2340 (M^+^, 100).

*10-(4-Methoxyphenylamino)-7,12-dimethyl-13-tetradecen-7-ol* (**1c**). (a mixture of major and minor isomers in a ratio of 3:1): IR (neat) 3379 (s), 2932 (s), 2359 (m), 1639 (s), 1514 (s), 1238 (s), 1043 (s), 912 (s), 818 (s) cm^−1^; ^1^H-NMR (CDCl_3_, major-isomer) δ 0.88 (t, *J* = 6.4 Hz, 3 H), 0.96 (d, *J* = 6.8 Hz, 3 H), 1.12 –1.26 (m, 8 H), 1.42 (br, 3 H), 1.49–1.69 (m, 8 H), 2.29 (qm, *J* = 6.8 Hz, 1 H), 3.25–3.33 (m, 1 H), 3.74 (s, 3 H), 4.91 (dd, *J* = 10.7, 1.5 Hz, 1 H), 4.92 (dd, *J* = 17.5, 1.5 Hz, 1 H), 5.63 (ddd, *J* = 17.5, 10.7, 8.0 Hz, 1 H), 6.69–6.72 (m, 2 H), 6.75 (d, *J* = 9.3 Hz, 2 H); ^13^C-NMR (CDCl_3_, major-isomer) δ 14.1, 20.7, 20.9, 22.6, 26.9, 27.6, 29.8, 31.8, 35.0, 37.4, 37.6, 41.9, 55.6, 55.7, 72.6, 113.3, 114.0, 116.8, 143.4, 143.9, 144.0; ^1^H-NMR (CDCl_3_, minor-isomer) δ 0.88 (t, *J* = 6.4 Hz, 3 H), 0.97 (d, *J* = 6.8 Hz, 3 H), 1.12 –1.26 (m, 8 H), 1.42 (br, 3 H), 1.49–1.69 (m, 8 H), 2.29 (qm, *J* = 6.8 Hz, 1 H), 3.25–3.33 (m, 1 H), 3.74 (s, 3 H), 4.91 (dd, *J* = 10.7, 1.5 Hz, 1 H), 4.92 (dd, *J* = 17.5, 1.5 Hz, 1 H), 5.63 (ddd, *J* = 17.5, 10.7, 8.0 Hz, 1 H), 6.69–6.72 (m, 2 H), 6.75 (d, *J* = 9.0 Hz, 2 H); ^13^C-NMR (CDCl_3_, minor-isomer) δ 14.1, 20.8, 20.9, 22.6, 26.8, 27.6, 29.8, 31.8, 35.0, 37.4, 37.6, 41.9, 55.6, 55.7, 72.7, 113.4, 114.0, 116.7, 143.5, 143.9, 144.0; HRMS, calcd for C_23_H_39_NO_2_: 361.2981. Found *m/z* (relative intensity): 361.2969 (M^+^, 100).

*5-[(4-Methoxyphenyl)amino]-7-methylnon-8-en-1-ol* (**1d**) (a mixture of major and minor isomers in a ratio of 6:1): IR (neat) 3368 (s), 2934 (s), 1514 (m), 1458 (s), 1236 (s), 1040 (s), 820 (s) cm^−1^; ^1^H-NMR (CDCl_3_, major-isomer) δ 1.00 (d, *J* = 6.6 Hz, 3 H), 1.37–1.58, (m, 8 H), 2.33 – 2.38 (m, 1 H), 3.31 (m, 1 H), 3.62 (t, *J* = 6.3 Hz, 2 H), 3.73 (s, 3 H), 4.92 (dd, *J* = 10.4, 1.1 Hz, 1 H), 4.93 (dd, *J* = 17.0, 1.1 Hz, 1 H), 5.65 (ddd, *J* = 17.0, 10.4, 8.1 Hz, 1 H), 6.52 (d, *J* = 8.9 Hz, 2 H), 6.74 (d, *J* = 8.9 Hz, 2 H); ^13^C-NMR (CDCl_3_, major-isomer) δ 21.1, 22.0, 32.8, 35.0, 35.2, 42.5, 52.1, 55.8, 62.8, 113.2, 114.4, 114.9, 142.1, 144.3, 151.6; ^1^H-NMR (CDCl_3_, minor-isomer) δ 0.98 (d, *J* = 6.8 Hz, 3 H), 1.37–1.58, (m, 8 H), 2.33–2.38 (m, 1 H), 3.31 (m, 1 H), 3.61 (t, *J* = 6.5 Hz, 2 H), 3.74 (s, 3 H), 4.92 (dd, *J* = 10.4, 1.1 Hz, 1 H), 4.93 (dd, *J* = 17.0, 1.1 Hz, 1 H), 5.65 (ddd, *J* = 17.0, 10.4, 8.1 Hz, 1 H), 6.52 (d, *J* = 8.9 Hz, 2 H), 6.74 (d, *J* = 8.9 Hz, 2 H); ^13^C-NMR (CDCl_3_, minor-isomer) δ 21.1, 21.9, 32.8, 35.0, 35.2, 42.1, 52.1, 55.8, 62.8, 113.2, 114.2, 114.8, 142.1, 144.3, 151.6; HRMS, calcd for C_17_H_27_ NO_2_: 277.2018. Found *m/z* (relative intensity): 278.2001 (M^+^+1, 1), 277.2014 (M^+^, 2), 260.1983 (13), 204.1406 (100).

*(5S,7S)-5-(4-Methoxyphenylamino)-3,3,7-trimethylnonen-8-ol* (**1e**). (a mixture of major and minor isomers in a ratio of 4:1): IR (neat) 3373 (s), 2932 (s), 2359 (m), 1732 (s), 1514 (s), 1234 (s), 1042 (s), 818 (s) cm^−1^; ^1^H-NMR (CDCl_3_, major-isomer) δ 0.94 (s, 6 H), 0.97 (d, *J* = 6.8 Hz, 3 H), 1.21–1.70(m, 6 H), 2.28 (qm, *J* = 6.8 Hz, 1 H), 3.02 (br, 1 H),3.34 (m, 1 H), 3.69 (s, 3 H), 3.66–3.76 (m, 2 H), 4.98 (dd, *J* = 17.7, 1.8 Hz, 1 H), 4.99 (dd, *J* = 10.0, 1.8 Hz, 1 H), 5.67 (ddd, *J* = 17.7, 10.0, 8.3 Hz, 1 H), 6.55 (br, 2 H), 6.75 (br d, *J* = 8.3 Hz, 2 H); ^13^C-NMR (CDCl_3_, major-isomer) δ 20.1, 21.6, 28.4, 28.5, 32.7, 44.5, 47.5, 47.9, 49.3, 55.0, 59.6, 113.6, 114.9, 115.0, 144.1, 144.7, 152.0; ^1^H-NMR (CDCl_3_, minor-isomer) δ 0.95 (s, 6 H), 0.99 (d, *J* = 6.6 Hz, 3 H), 1.21–1.70 (m, 6 H), 2.28 (qm, *J* = 6.8 Hz, 1 H), 3.02 (br, 1 H),3.34 (m, 1 H), 3.67 (s, 3 H), 3.66–3.76 (m, 2 H), 4.92 (dd, *J* = 10.2, 0.8 Hz, 1 H), 4.95 (dd, *J* = 16.9, 0.8 Hz, 1 H), 5.67 (ddd, *J* = 17.7, 10.0, 8.3 Hz, 1 H), 6.55 (br, 2 H), 6.75 (br d, *J* = 8.3 Hz, 2 H); ^13^C-NMR (CDCl_3_, minor-isomer) δ 20.1, 21.6, 28.4, 28.5, 32.6, 44.3, 47.5, 47.9, 49.3, 55.0, 59.6, 113.6, 114.8, 115.0, 144.1, 144.7, 152.0; HRMS, calcd for C_19_H_31_NO_2_: 305.2355. Found *m/z* (relative intensity): 306.2373 (M^+^+1, 9), 305.2337 (M^+^, 44), 237.1655 (18), 236.1608 (100), 235.1535 (19).

*2-((3S,5S)-3-(4-Methoxyphenylamino)-5-methyl-6-heptenyl)phenol* (**1f**). (a mixture of major and minor isomers in a ratio of 7:1): IR (neat) 3308 (s), 2930 (s), 1583 (m), 1506 (s), 1236 (s), 1040 (s), 822 (s), 754 (s) cm^−1^; ^1^H-NMR (CDCl_3_, major-isomer) δ 0.84 (d, *J* = 6.8 Hz, 3 H), 1.11–1.68 (m, 4 H), 2.20 (qm, *J* = 6.8 Hz, 1 H), 2.65–2.71 (m, 2 H), 2.88–2.95 (m, 1 H), 3.13–3.29 (m, 1 H), 3.75 (s, 3 H), 4.86 (dd, *J* = 10.8, 0.8 Hz, 1 H), 4.87 (dd, *J* = 16.7, 0.8 Hz, 1 H), 5.57 (ddd, *J* = 16.7, 10.8, 7.8 Hz, 1 H), 6.78 (d, *J* = 8.6 Hz, 2 H), 6.84 (d, *J* = 8.6 Hz, 2 H), 6.73–6.92 (m, 2 H), 7.06–7.12 (m, 2 H); ^13^C-NMR (CDCl_3_, major-isomer) δ 20.2, 26.2, 34.8, 35.3, 40.0, 53.3, 55.6, 55.7, 112.9, 114.8, 116.3, 120.3, 127.2, 127.3, 127.4, 129.9, 130.0, 144.3, 154.8; ^1^H-NMR (CDCl_3_, minor-isomer) δ 0.89 (d, *J* = 6.3 Hz, 3 H), 1.11–1.68 (m, 4 H), 2.20 (qm, *J* = 6.8 Hz, 1 H), 2.65–2.71 (m, 2 H), 2.88–2.95 (m, 1 H), 3.13–3.29 (m, 1 H), 3.75 (s, 3 H), 4.86 (dd, *J* = 10.8, 0.8 Hz, 1 H), 4.87 (dd, *J* = 16.7, 0.8 Hz, 1 H), 5.57 (ddd, *J* = 16.7, 10.8, 7.8 Hz, 1 H), 6.78 (d, *J* = 8.6 Hz, 2 H), 6.84 (d, *J* = 8.6 Hz, 2 H), 6.73–6.92 (m, 2 H), 7.06–7.12 (m, 2 H); ^13^C-NMR (CDCl_3_, minor-isomer) δ 20.2, 26.2, 34.8, 35.3, 40.0, 53.3, 55.6, 55.7, 112.9, 114.6, 116.3, 120.6, 127.2, 127.3, 127.4, 129.9, 130.1, 144.3, 154.8; HRMS, calcd for C_21_H_27_NO_2_: 325.2042. Found *m/z* (relative intensity): 326.2086 (M^+^+1, 18), 325.2045 (M^+^, 78), 257.1349 (18), 256.1329 (100).

*Tert-Butyl(5S,7S)-5-(4-methoxyphenylamino)-7-methylnon-8-enylcarbamate* (**1g**). (a mixture of major and minor isomers in a ratio of 3:1):IR (neat) 2864 (s), 2359 (m), 1682 (s), 1539 (s), 1251 (s), 1173 (s), 910 (s), 750 (s) cm^−1^; ^1^H-NMR (CDCl_3_, major-isomer) δ 1.01 (d, *J* = 6.8 Hz, 3 H), 1.34–1.51 (m, 8 H), 1.44 (s, 9 H), 2.32 (dm, *J* = 7.4 Hz, 1 H), 3.11 (br, 2 H), 3.67 (br, 1 H), 4.93 (dd, *J* = 10.2, 1.1 Hz, 1 H), 5.01 (dd, *J* = 17.4, 1.1 Hz, 1 H), 5.77 (ddd, *J* = 17.4, 10.2, 7.4 Hz, 1 H); ^13^C-NMR (CDCl_3_, major-isomer) δ 20.2, 22.6, 28.4, 30.1, 35.4, 37.2, 40.4, 44.5, 70.1, 79.0, 112.6, 114.9, 155.9; ^1^H-NMR (CDCl_3_, minor-isomer) δ 0.98 (d, *J* = 6.6 Hz, 3 H), 1.34–1.51 (m, 8 H), 1.44 (s, 9 H), 2.32 (dm, *J* = 7.4 Hz, 1 H), 3.11 (br, 2 H), 3.67 (br, 1 H), 4.88 (dm, *J* = 11.2 Hz, 1 H), 5.01 (dd, *J* = 17.4, 1.1 Hz, 1 H), 5.77 (ddd, *J* = 17.4, 10.2, 7.4 Hz, 1 H); ^13^C-NMR (CDCl_3_, minor-isomer) δ 19.8, 22.6, 28.4, 30.0, 35.4, 37.2, 40.4, 44.3, 69.8, 79.0, 112.0, 114.6, 155.9; HRMS, calcd for C_15_H_29_NO_3_: 271.2147. Found *m/z* (relative intensity): 376.2731 (M^+^, 100).

*(6S,8S)-6-(4-Methoxyphenylamino)-8-methyldec-9-en-1-ol* (**1h**). (a mixture of major and minor isomers in a ratio of 4:1): IR (neat) 3364 (s), 2934 (s), 1614 (m), 1514 (s), 1238 (s), 1038 (s), 822 (s) cm^−1^; ^1^H-NMR (CDCl_3_, major-isomer) δ 1.00 (d, *J* = 6.8 Hz, 3 H), 1.21–1.58, (m, 10 H), 2.34–2.38 (m, 1 H), 3.27–3.32 (m, 1 H), 3.61 (t, *J* = 6.6 Hz, 2 H), 3.74 (s, 3 H), 4.92 (dd, *J* = 10.2, 0.9 Hz, 1 H), 4.93 (dd, *J* = 17.0, 0.9 Hz, 1 H), 5.65 (ddd, *J* = 17.0, 10.2, 8.1 Hz, 1 H), 6.63 (d, *J* = 9.1 Hz, 2 H), 6.74 (d, *J* = 9.1 Hz, 2 H); ^13^C-NMR (CDCl_3_, major-isomer) δ 21.2, 25.6, 25.9, 32.8, 35.1, 35.3, 42.4, 55.8, 55.9, 62.9, 113.3, 114.9, 116.4, 139.8, 144.3, 152.8; ^1^H-NMR (CDCl_3_, minor-isomer) δ 0.97 (d, *J* = 6.8 Hz, 3 H), 1.21–1.58, (m, 10 H), 2.34–2.38 (m, 1 H), 3.27–3.32 (m, 1 H), 3.61 (t, *J* = 6.4 Hz, 2 H), 3.74 (s, 3 H), 4.92 (dd, *J* = 10.2, 0.9 Hz, 1 H), 4.93 (dd, *J* = 17.0, 0.9 Hz, 1 H), 5.65 (ddd, *J* = 17.0, 10.2, 8.1 Hz, 1 H), 6.63 (d, *J* = 9.1 Hz, 2 H), 6.74 (d, *J* = 9.1 Hz, 2 H); ^13^C-NMR (CDCl_3_, minor-isomer) δ 21.2, 25.6, 25.9, 32.8, 35.1, 35.3, 42.4, 55.8, 55.9, 63.1, 113.3, 114.8, 116.4, 139.8, 144.3, 152.8; HRMS, calcd for C_18_H_29_NO_2_: 291.2196. Found *m/z* (relative intensity): 292.2236 (M^+^+1, 15), 291.2196 (M^+^, 65), 223.1501 (13), 222.1497 (100).

*2-(4-Methoxyphenylamino)-5-hexenol* (**1i**). IR (neat) 3375 (s), 3076 (m), 2936 (s), 1639 (s), 1514 (s), 1464 (s), 1238 (s), 1038 (s), 822 (s) cm^−1^; ^1^H-NMR (CDCl_3_) δ 1.42 (quin, *J* = 7.5 Hz, 2 H), 2.14 (br q, *J* = 6.8 Hz, 1 H), 2.25 (br q, *J* = 6.4 Hz, 1 H), 2.69 (br, 1 H), 3.40 (m, 2 H), 3.49 (dd, *J* = 10.9, 6.1 Hz, 1 H), 3.51 (dd, *J* = 10.9, 6.1 Hz, 1 H), 3.74 (s, 3 H), 4.94 (dm, *J* = 9.7 Hz, 1 H), 5.00 (dt, *J* = 16.1,1.9 Hz, 1 H), 5.78 (ddt, *J* = 16.1, 9.7, 6.4 Hz, 1 H), 6.64 (dd, *J* = 6.6, 2.4 Hz, 2 H), 6.74 (dd, *J* = 6.6, 2.4 Hz, 2 H); ^13^C-NMR (CDCl_3_) δ 28.7, 30.4, 55.8, 56.3, 64.1, 114.8, 115.7, 116.4, 137.8, 139.8, 152.7; HRMS, calcd for C_13_H_19_NO_2_: 221.1416. Found *m/z* (relative intensity): 222.1449 (M^+^+1, 4), 221.1401 (M^+^, 28), 191.1235 (14), 190.1195 (100).

*(E)-2-(4-Methoxyphenylamino)-4-hexenol* (**1i'**). ^1^H-NMR (CDCl_3_) δ 1.64 (dm, *J* = 7.6 Hz, 3 H), 2.02 (m, 2 H), 3.40 (m, 2 H), 3.49 (dd, *J* = 10.9, 6.1 Hz, 1 H), 3.51 (dd, *J* = 10.9, 6.1 Hz, 1 H), 3.74 (s, 3 H), 5.40 (m, 1 H), 5.50 (m, 1 H), 6.64 (dd, *J* = 6.6, 2.4 Hz, 2 H), 6.74 (dd, *J* = 6.6, 2.4 Hz, 2 H); ^13^C-NMR (CDCl_3_) δ 18.0, 33.3, 55.8, 56.5, 64.4, 114.9, 116.1, 125.7, 133.8, 139.8, 152.8.

*(2R,4S)-4-Methyl-2-(phenylamino)-5-hexenol* (**1j**). (a mixture of major and minor isomers in a ratio of 8:1): IR (neat) 3393 (s), 3078 (m), 2926 (s), 1601 (s), 1506 (s), 1317 (s), 1030 (s), 914 (m), 748 (s), 692 (s) cm^−1^; ^1^H-NMR (CDCl_3_, major-isomer) δ 1.03 (d, *J* = 6.8 Hz, 3 H), 1.52 (t, *J* = 5.7 Hz, 2 H), 2.32 (ddm, *J* = 7.7, 6.8 Hz, 1 H), 3.49 (dd, *J* = 10.5, 5.4 Hz, 1 H), 3.55 (tdm, *J* = 5.4, 4.1 Hz, 2 H), 3.71 (dd, *J* = 10.5, 4.1 Hz, 1 H), 4.89 (dd, *J* = 17.2, 0.9 Hz, 1 H), 4.92 (dd, *J* = 10.4, 0.9 Hz, 1 H), 5.64 (ddd, *J* = 17.2, 10.4, 7.7 Hz, 1 H), 6.64 (dd, *J* = 8.6, 1.2 Hz, 2 H), 6.70 (t, *J* = 7.3 Hz, 1 H), 7.15 (dd, *J* = 8.6, 7.3 Hz, 2 H); ^13^C-NMR (CDCl_3_, major-isomer) δ 21.1, 35.0, 39.8, 53.5, 65.0, 113.7, 113.8, 117.8, 129.3, 143.7, 147.7; ^1^H-NMR (CDCl_3_, minor-isomer) δ 1.00 (d, *J* = 6.8 Hz, 3 H), 1.50 (t, *J* = 5.1 Hz, 2 H), 2.32 (ddm, *J* = 7.7, 6.8 Hz, 1 H), 3.49 (dd, *J* = 10.5, 5.4 Hz, 1 H), 3.55 (tdm, *J* = 5.4, 4.1 Hz, 2 H), 3.71 (dd, *J* = 10.5, 4.1 Hz, 1 H), 4.89 (dd, *J* = 17.2, 0.9 Hz, 1 H), 4.92 (dd, *J* = 10.4, 0.9 Hz, 1 H), 5.64 (ddd, *J* = 17.2, 10.4, 7.7 Hz, 1 H), 6.64 (dd, *J* = 8.6, 1.2 Hz, 2 H), 6.70 (t, *J* = 7.3 Hz, 1 H), 7.14 (dd, *J* = 8.5, 7.3 Hz, 2 H); ^13^C-NMR (CDCl_3_, minor-isomer) δ 21.1, 35.0, 39.8, 53.5, 65.0, 113.7, 113.8, 117.8, 129.3, 143.7, 147.7; HRMS, calcd for C_13_H_19_NO: 205.1467. Found *m/z* (relative intensity): 206.1505 (M^+^+1, 4), 205.1463 (M^+^, 20), 175.1278 (16), 174.1268 (100).

*(2R,4S)-2-(4-Methoxyphenylamino)-4-methyl-5-hexenol* (**1k**). IR (neat) 3383 (s), 3078 (m), 2932 (s), 1618 (s), 1418 (s), 1238 (s), 1040 (s), 914 (m), 820 (s), 667 (m) cm^−1^; ^1^H-NMR (CDCl_3_) δ 1.01 (d, *J* = 6.6 Hz, 3 H), 1.49 (t, *J* = 6.8 Hz, 2 H), 2.31 (qtm, *J* = 6.8, 6.6 Hz, 1 H), 3.45 (dd, *J* = 8.4, 3.2 Hz, 1 H), 3.46 (dd, *J* = 8.4, 5.5 Hz, 1 H), 3.71 (dm, *J* = 6.6 Hz, 1 H), 3.74 (s, 3 H), 4.89 (dd, *J* = 17.1, 1.2 Hz, 1 H), 4.92 (dd, *J* = 10.2, 1.2 Hz, 1 H), 5.63 (ddd, *J* = 17.1, 10.2, 8.1 Hz, 1 H), 6.64 (dd, *J* = 6.6, 2.2 Hz, 2 H), 6.76 (dd, *J* = 6.6, 2.2 Hz, 2 H); ^13^C-NMR (CDCl_3_) δ 21.0, 34.9, 39.6, 55.0, 55.7, 64.8, 113.6, 114.8, 115.4, 141.7, 143.7, 152.3; HRMS, calcd for C_14_H_21_NO_2_: 235.1572. Found *m/z* (relative intensity): 236.1583 (M^+^+1, 5), 235.1562 (M^+^, 28), 204.1378 (100).

*(2R,4S)-2-(2-Methoxyphenylamino)-4-methyl-5-hexenol* (**1l**). (a mixture of major and minor isomers in a ratio of 8:1): IR (neat) 3414 (s), 3070 (m), 2932 (s), 2359 (s), 1601 (s), 1516 (s), 1456 (s), 1223 (s), 1030 (s), 914 (s), 737 (s) cm^−1^; ^1^H-NMR (CDCl_3_, major-isomer) δ 1.03 (d, *J* = 6.8 Hz, 3 H), 1.54 (t, *J* = 6.6 Hz, 2 H), 2.31 (qm, *J* = 6.8 Hz, 1 H), 3.50 (dd, *J* = 10.4, 5.8 Hz, 1 H), 3.55 (tdm, *J* = 6.6, 3.9 Hz, 1 H), 3.72 (dd, *J* = 10.4, 3.9 Hz, 1 H), 3.85 (s, 3 H), 4.87 (dd, *J* = 17.8, 1.7 Hz, 1 H), 4.90 (dd, *J* = 10.1, 1.7 Hz, 1 H), 5.64 (ddd, *J* = 17.8, 10.1, 8.0 Hz, 1 H), 6.68 (d, *J* = 7.6 Hz, 1 H), 6.69 (dd, *J* = 7.6, 1.6 Hz, 1 H), 6.77 (dd, *J* = 7.6, 1.2 Hz, 1 H), 6.83 (dm, *J* = 7.6 Hz, 1 H); ^13^C-NMR (CDCl_3_, major-isomer) δ 21.1, 34.9, 39.8, 53.4, 55.5, 65.1, 109.8, 111.2, 113.7, 116.9, 121.3, 137.4, 143.7, 147.0; ^1^H-NMR (CDCl_3_, minor-isomer) δ 0.99 (d, *J* = 6.8 Hz, 3 H), 1.54 (t, *J* = 6.6 Hz, 2 H), 2.31 (qm, *J* = 6.8 Hz, 1 H), 3.50 (dd, *J* = 10.4, 5.8 Hz, 1 H), 3.55 (tdm, *J* = 6.6, 3.9 Hz, 1 H), 3.72 (dd, *J* = 10.4, 3.9 Hz, 1 H), 3.85 (s, 3 H), 4.87 (dd, *J* = 17.8, 1.7 Hz, 1 H), 4.90 (dd, *J* = 10.1, 1.7 Hz, 1 H), 5.64 (ddd, *J* = 17.8, 10.1, 8.0 Hz, 1 H), 6.68 (d, *J* = 7.6 Hz, 1 H), 6.69 (dd, *J* = 7.6, 1.6 Hz, 1 H), 6.77 (dd, *J* = 7.6, 1.2 Hz, 1 H), 6.83 (dm, *J* = 7.6 Hz, 1 H); ^13^C-NMR (CDCl_3_, minor-isomer) δ 21.1, 35.0, 39.8, 53.4, 55.6, 65.1, 109.8, 111.2, 113.7, 116.9, 121.3, 137.4, 143.8, 147.0; HRMS, calcd for C_14_H_21_NO_2_: 235.1572. Found *m/z* (relative intensity): 235.1568 (M^+^, 29), 205.1415 (19), 204.1378 (100).

*(2R,4S)-2-(4-Bromophenylamino)-4-methyl-5-hexenol* (**1m**). IR (neat) 3400 (s), 2927 (s), 2868 (s), 2362 (m), 2345 (s), 1593 (s), 1496 (s), 1317 (s), 1074 (s), 916 (m), 812 (s) cm^−1^; ^1^H-NMR (CDCl_3_) δ1.01 (d, *J* = 6.6 Hz, 3 H), 1.50 (m, 2 H), 2.02 (br, 1 H), 2.30 (m, 1 H), 3.48 (m, 2 H), 3.71 (dd, *J* = 13 6.2 Hz, 1 H), 4.88 (dd, *J* = 23, 1.2 Hz, 1 H), 4.92 (dd, *J* = 16, 1.2 Hz, 1 H), 5.62 (ddd, *J* = 17.0, 10.2, 8.1 Hz, 1 H), 6.49 (d, *J* = 9.0 Hz, 2 H), 7.21 (d, *J* = 9.0 Hz, 2 H); ^13^C-NMR (CDCl_3_) δ 21.0, 34.9, 38.5, 53.4, 64.8, 109.0, 113.9, 115.0, 131.8, 143.5, 146.7; HRMS, calcd for C_13_H_18_BrNO: 283.0572. Found *m/z* (relative intensity): 283.0562 (M^+^, 25), 254.0476 (100).

*(2R,4S)-2-(4-Methoxyphenylamino)-8-methyl-4-vinylnon-7-enol* (**1n**). IR (neat) 3368 (s), 3078 (m), 2916 (s), 1607 (m), 1514 (s), 1375 (s), 1240 (s), 1042 (s), 914 (s), 820 (s) cm^−1^; ^1^H-NMR (CDCl_3_) δ 1.33 (m, 2 H), 1.46 (dd, *J* = 10.1, 4.4 Hz, 2 H), 1.56 (s, 3 H), 1.67 (s, 3 H), 1.94 (m, 2 H), 2.15 (dm, *J* = 4.4 Hz, 1 H), 3.44 (dd, *J* = 10.0, 7.4 Hz, 1 H), 3.46 (dd, *J* = 10.0, 5.8 Hz, 1 H), 3.69 (m, 2 H), 3.74 (s, 3 H), 4.84 (dd, *J* = 17.0, 2.0 Hz, 1 H), 4.99 (dd, *J* = 10.2, 2.0 Hz, 1 H), 5.05 (tt, *J* = 5.6, 1.4 Hz, 1 H), 5.49 (ddd, *J* = 17.0, 10.2, 9.0 Hz, 1 H), 6.65 (dd, *J* = 6.6, 2.3 Hz, 2 H), 6.75 (dd, *J* = 6.6, 2.3 Hz, 2 H); ^13^C-NMR (CDCl_3_) δ 17.7, 25.6, 25.7, 35.5, 38.1, 40.5, 55.7, 65.0, 114.8, 115.5, 115.8, 124.1, 131.4, 141.2, 142.2, 152.7; HRMS, calcd for C_19_H_29_NO_2_: 303.2198. Found *m/z* (relative intensity): 304.2201 (M^+^+1, 11), 303.2183 (M^+^, 48), 273.2022 (19), 272.2000 (100).

*3-(4-Methoxyphenylamino)-6-hexene-1,2-diol* (**1o**). (a mixture of major and minor isomers in a ratio of 3:1): IR (neat) 3356 (s), 3074 (m), 2934 (s), 1666 (s), 1441 (s), 1236 (s), 1038 (s), 822 (s) cm^−1^; ^1^H-NMR (CDCl_3_, major-isomer) δ 1.62 (td, *J* = 8.7, 5.9 Hz, 2 H), 2.00–2.33 (m, 2 H), 2.60–3.00 (m, 1 H), 3.35–3.47 (m, 1 H), 3.68–3.80 (m, 2 H), 3.74 (s, 3 H), 4.95 (dd, *J* = 9.9, 1.5 Hz, 1 H), 4.96 (dd, *J* = 17.8, 1.5 Hz, 1 H), 5.76 (ddt, *J* = 17.8, 9.9, 6.7 Hz, 1 H), 6.65 (dt, *J* = 9.3, 2.5 Hz, 2 H), 6.76 (dt, *J* = 9.3, 2.5 Hz, 2 H); ^13^C-NMR (CDCl_3_, major-isomer) δ 30.3, 30.8, 55.7, 57.8, 64.0, 72.7, 114.9, 115.2, 115.6, 137.7, 141.1, 152.6; ^1^H-NMR (CDCl_3_, minor-isomer) δ 1.62 (td, *J* = 8.7, 5.9 Hz, 2 H), 2.00–2.33 (m, 2 H), 2.60–3.00 (m, 1 H), 3.35–3.47 (m, 1 H), 3.68–3.80 (m, 2 H), 3.74 (s, 3 H), 4.95 (dd, *J* = 9.9, 1.5 Hz, 1 H), 4.96 (dd, *J* = 17.8, 1.5 Hz, 1 H), 5.76 (ddt, *J* = 17.8, 9.9, 6.7 Hz, 1 H), 6.65 (dt, *J* = 9.3, 2.5 Hz, 2 H), 6.76 (dt, *J* = 9.3, 2.5 Hz, 2 H); ^13^C-NMR (CDCl_3_, minor-isomer) δ 30.2, 30.8, 55.7, 57.8, 64.2, 72.6, 114.8, 115.2, 115.6, 137.7, 141.2, 152.6; HRMS, calcd for C_14_H_21_NO_3_: 251.1521. Found *m/z* (relative intensity): 252.1614 (M^+^+1, 4), 251.1512 (M^+^, 30), 220.1334 (6), 191.1258 (14), 190.1187 (100).

*(E)-3-(4-Methoxyphenylamino)-5-hexene-1,2-diol* (**1o'**). (a mixture of major and minor isomers in a ratio of 3:1): ^1^H-NMR (CDCl_3_, major-isomer) δ 1.72 (d, *J* = 6.8 Hz, 3 H), 2.00–2.33 (m, 2 H), 2.60–3.00 (m, 1 H), 3.35–3.47 (m, 1 H), 3.68–3.80 (m, 2 H), 3.74 (s, 3 H), 5.36–5.43 (m, 1 H), 5.48–5.55, (m, 1 H), 6.65 (dt, *J* = 9.3, 2.5 Hz, 2 H), 6.76 (dt, *J* = 9.3, 2.5 Hz, 2 H); ^13^C-NMR (CDCl_3_, major-isomer) δ 18.0, 33.8, 55.7, 57.4, 64.8, 72.2, 114.9, 115.2, 126.5, 128.8, 141.5, 152.8; ^1^H-NMR (CDCl_3_, minor-isomer) δ 1.72 (d, *J* = 6.8 Hz, 3 H), 2.00–2.33 (m, 2 H), 2.60–3.00 (m, 1 H), 3.35–3.47 (m, 1 H), 3.68–3.80 (m, 2 H), 3.74 (s, 3 H), 5.36–5.43 (m, 1 H), 5.48–5.55, (m, 1 H), 6.65 (dt, *J* = 9.3, 2.5 Hz, 2 H), 6.76 (dt, *J* = 9.3, 2.5 Hz, 2 H); ^13^C-NMR (CDCl_3_, minor-isomer) δ 18.0, 33.8, 55.7, 57.4, 64.7, 72.2, 114.8, 115.2, 126.5, 128.8, 141.5, 152.8.

*(3R,5S)-5-Methyl-3-(phenylamino)-6-heptene-1,2-diol* (**1p**). (a mixture of major and minor isomers in a ratio of 2:1): IR (neat) 3281 (s), 2961 (s), 1603 (s), 1512 (s), 1325 (s), 1024 (s), 748 (s), 692 (s) cm^−1^; ^1^H-NMR (CDCl_3_, major-isomer) δ 1.01 (d, *J* = 6.8 Hz, 3 H), 1.54–1.63 (m, 2 H), 2.23–2.33 (qm, *J* = 6.8 Hz, 1 H), 3.54–3.77 (m, 4 H), 4.94 (dd, *J* = 10.3, 1.5 Hz, 1 H), 4.98 (dd, *J* = 17.1, 1.5 Hz, 1 H), 5.59 (ddd, *J* = 17.1, 10.3, 8.3 Hz, 1 H), 6.65 (td, *J* = 8.5, 1.0 Hz, 2 H), 6.70–6.74 (m, 1 H), 7.15 (dt, *J* = 8.5, 7.6 Hz, 2 H); ^13^C-NMR (CDCl_3_, major-isomer) δ 21.4, 34.9, 39.0, 54.3, 64.0, 73.5, 113.7, 117.8, 118.1, 129.2, 143.4, 147.6; ^1^H-NMR (CDCl_3_, minor-isomer) δ 0.97 (d, *J* = 6.6 Hz, 3 H), 1.47 (ddd, *J* = 14.0, 9.8, 4.2 Hz, 2 H), 2.23–2.33 (qm, *J* = 6.8 Hz, 1 H), 3.54–3.77 (m, 4 H), 4.82 (dd, *J* = 17.3, 1.7 Hz, 1 H), 4.92 (dd, *J* = 9.9, 1.7 Hz, 1 H), 5.72 (ddd, *J* = 17.3, 9.9, 7.4 Hz, 1 H), 6.65 (td, *J* = 8.5, 1.0 Hz, 2 H), 6.70–6.74 (m, 1 H), 7.15 (dt, *J* = 8.5, 7.6 Hz, 2 H); ^13^C-NMR (CDCl_3_, minor-isomer) δ 21.2, 34.5, 38.2, 54.6, 63.8, 72.7, 113.8, 117.8, 118.0, 129.3, 143.9, 147.4; HRMS, calcd for C_14_H_21_NO_2_: 235.1572. Found *m/z* (relative intensity): 236.1596 (M^+^+1, 3), 235.1554 (M^+^, 12), 175.1262 (13), 174.1240 (100).

*(3R,5S)-3-(4-Methoxyphenylamino)-5-methyl-6-heptene-1,2-diol* (**1q**). (a mixture of major and minor isomers in a ratio of 2:1): IR (neat) 3366 (s), 3078 (m), 2932 (m), 2835 (m), 1655 (s), 1238 (s), 1036 (s), 822 (s) cm^−1^; ^1^H-NMR (CDCl_3_, major-isomer) δ 1.00 (d, *J* = 6.6 Hz, 3 H), 1.48 (ddd, *J* = 14.0, 9.7, 4.2 Hz, 1 H), 1.55 (ddd, *J* = 14.0, 9.7, 4.2 Hz, 1 H), 2.03 (br, 2 H) 2.25 (m, 1 H), 3.44–3.54 (m, 1 H), 3.74 (s, 3 H), 3.66–3.8 (m, 4 H), 4.80 (dd, *J* = 17.1, 1.3 Hz, 1 H), 4.91 (dd, *J* = 10.5, 1.3 Hz, 1 H), 5.57 (ddd, *J* = 17.1, 10.5, 8.3 Hz, 1 H), 6.68 (dd, *j* = 6.8, 2.3 Hz, 2 H), 6.76 (dd, *J* = 6.8, 2.3 Hz, 2 H); ^13^C-NMR (CDCl_3_, major-isomer) δ 21.4, 35.0, 39.0, 55.8, 56.3, 64.1, 73.1, 114.2, 114.9, 115.9, 143.5, 143.7, 152.8; ^1^H-NMR (CDCl_3_, minor-isomer) δ 0.96 (d, *J* = 6.6 Hz, 3 H), 1.48 (ddd, *J* = 14.0, 9.7, 4.2 Hz, 1 H), 1.55 (ddd, *J* = 14.0, 9.7, 4.2 Hz, 1 H), 2.03 (br, 2 H) 2.25 (m, 1 H), 3.44–3.54 (m, 1 H), 3.74 (s, 3 H), 3.66–3.8 (m, 4 H), 4.80 (dd, *J* = 17.1, 1.3 Hz, 1 H), 4.97 (dd, *J* = 17.4, 1.3 Hz, 1 H), 5.67 (ddd, *J* = 17.4, 10.2, 7.6 Hz, 1 H), 6.68 (dd, *J* = 6.8, 2.3 Hz, 2 H), 6.76 (dd, *J* = 6.8, 2.3 Hz, 2 H); ^13^C-NMR (CDCl_3_, minor-isomer) δ 20.4, 34.7, 39.0, 55.8, 56.3, 63.8, 73.1, 115.0, 114.9, 116.1, 143.5, 143.7, 152.8; HRMS, calcd for C_15_H_23_NO_3_: 265.1678. Found *m/z* (relative intensity): 266.1678 (M^+^+1, 3), 265.1659 (M^+^, 18), 205.1406 (15), 204.1406 (100).

*(3R,5S)-3-(4-Methoxyphenylamino)-9-methyl-5-vinyl-8-decene-1,2-diol* (**1r**). (a mixture of major and minor isomers in a ratio of 2:1): IR (neat) 3358 (s), 3074 (s), 2916 (s), 2343 (m), 1666 (s), 1514 (s), 1238 (s), 1040 (s), 822 (s) cm^−1^; ^1^H-NMR (CDCl_3_, major-isomer) δ 1.19–1.38 (m, 4 H), 1.55 (s, 3 H), 1.67 (s, 3 H), 1.79–1.98 (dm, *J* = 7.5 Hz, 1 H), 2.00–2.17 (m, 2 H), 3.47 (dd, *J* = 12.0, 5.9 Hz, 1 H), 3.54 (dd, *J* = 12.0, 6.9 Hz, 1 H), 3.69–3.80 (m, 3 H), 3.74 (s, 3 H), 4.74 (dd, *J* = 18.6, 2.0 Hz, 1 H), 4.94–5.05 (m, 1 H), 4.98 (dd, *J* = 10.0, 2.0 Hz, 1 H), 5.46 (ddd, *J* = 18.6, 10.0, 7.5 Hz, 1 H), 6.69 (d, *J* = 6.6 Hz, 2 H), 6.75–6.78 (m, 2 H); ^13^C-NMR (CDCl_3_, major-isomer) δ 17.7, 25.4, 25.6, 35.2, 35.4, 40.5, 55.6, 55.7, 64.0, 73.1, 114.9, 115.3, 116.2, 124.1, 124.3, 131.5, 131.6, 141.9; ^1^H-NMR (CDCl_3_, minor-isomer) δ 1.19–1.38 (m, 4 H), 1.54 (s, 3 H), 1.64 (s, 3 H), 1.79–1.98 (dm, *J* = 7.5 Hz, 1 H), 2.00–2.17 (m, 2 H), 3.47 (dd, *J* = 12.0, 5.9 Hz, 1 H), 3.54 (dd, *J* = 12.0, 6.9 Hz, 1 H), 3.69–3.80 (m, 3 H), 3.75 (s, 3 H), 4.74 (dd, *J* = 18.6, 2.0 Hz, 1 H), 4.94–5.05 (m, 1 H), 4.98 (dd, *J* = 10.0, 2.0 Hz, 1 H), 5.46 (ddd, *J* = 18.6, 10.0, 7.5 Hz, 1 H), 6.69 (d, *J* = 6.6 Hz, 2 H), 6.75–6.78 (m, 2 H); ^13^C-NMR (CDCl_3_, minor-isomer) δ 17.7, 25.4, 25.6, 35.2, 35.4, 40.4, 55.6, 55.7, 64.0, 73.1, 114.8, 115.3, 116.3, 124.1, 124.3, 131.4, 131.6, 141.8; HRMS, calcd for C_20_H_31_NO_3_: 333.2304. Found *m/z* (relative intensity): 334.2348 (M^+^+1, 7), 333.2312 (M^+^, 33), 302.2102 (4), 273.2012 (20), 272.2004 (100).

*(2S, 3S, 5S)-5-(4-Methoxyphenylamino)-8-nonen-1,2,3-triol* (**1s**). (a mixture of major and minor isomers in a ratio of 1:1): IR (neat) 3277 (m), 2932 (s), 2839 (s), 1732 (s), 1514 (s), 1456 (s), 1441 (s), 1238 (s), 1040 (s), 970 (s), 912 (m), 824 (s) cm^−1^; ^1^H-NMR (CDCl_3_, major-isomer) δ 1.43 (m, 2 H), 1.87 (dt, *J* = 11.5, 3.2 Hz, 2 H), 2.15–2.30 (br d, *J* = 6.8 Hz, 2 H), 3.51–3.62 (m, 4 H), 3.61 (br t, *J* = 3.2 Hz, 1 H), 3.74 (s, 3 H), 3.93–4.01 (m, 1 H), 4.95 (dd, *J* = 11.2, 1.6 Hz, 1 H), 4.98 (dd, *J* = 18.1, 1.6 Hz, 1 H), 5.78 (ddd, *J* = 18.1, 11.2, 6.8 Hz, 1 H), 6.70 (d, *J* = 8.8 Hz, 2 H), 6.77 (d, *J* = 8.8 Hz, 2 H); ^13^C-NMR (CDCl_3_, major-isomer) δ 28.7, 32.7, 36.5, 55.7, 56.8, 63.7, 71.3, 74.6, 114.5, 114.8, 118.3, 138.5, 141.1, 153.2; ^1^H-NMR (CDCl_3_, minor-isomer) δ 1.43 (m, 2 H), 1.87 (dt, *J* = 11.5, 3.2 Hz, 2 H), 2.15–2.30 (br d, *J* = 6.8 Hz, 2 H), 3.51–3.62 (m, 4 H), 3.61 (br t, *J* = 3.2 Hz, 1 H), 3.74 (s, 3 H), 3.93–4.01 (m, 1 H), 4.95 (dd, *J* = 11.2, 1.6 Hz, 1 H), 4.98 (dd, *J* = 18.1, 1.6 Hz, 1 H), 5.78 (ddd, *J* = 18.1, 11.2, 6.8 Hz, 1 H), 6.70 (d, *J* = 8.8 Hz, 2 H), 6.77 (d, *J* = 8.8 Hz, 2 H); ^13^C-NMR (CDCl_3_, minor-isomer) δ 28.7, 32.7, 36.1, 55.7, 56.8, 63.7, 71.3, 74.7, 114.6, 114.8, 118.5, 138.5, 141.1, 153.3; HRMS, calcd for C_16_H_25_NO_4_: 295.1783. Found *m*/*z* (relative intensity): 296.1817 (M^+^+1, 21), 295.1776 (M^+^, 100), 294.1700 (5).

*(2S, 3S, 5S)-5-(4-Methoxyphenylamino)-7-nonen-1,2,3-triol* (**1s'**). (a mixture of major and minor isomers in a ratio of 3:1): ^1^H-NMR (CDCl_3_, major-isomer) δ 1.43 (m, 2 H), 1.87 (dt, *J* = 11.5, 3.2 Hz, 2 H), 2.15–2.30 (m, 2 H), 2.03 (d, *J* = 7.3 Hz, 3 H), 3.51–3.62 (m, 4 H), 3.61 (br t, *J* = 3.2 Hz, 1 H), 3.74 (s, 3 H), 3.93–4.01 (m, 1 H), 5.32 (dq, *J* = 14.6, 7.3 Hz, 1 H), 5.46 (dt, *J* = 14.6, 6.6 Hz, 1 H), 6.70 (d, *J* = 8.8 Hz, 2 H), 6.77 (d, *J* = 8.8 Hz, 2 H); ^13^C-NMR (CDCl_3_, major-isomer) δ 18.0, 32.5, 37.7, 52.7, 55.7, 63.9, 71.1, 74.1, 114.9, 116.9, 125.5, 130.6, 140.8, 154.0; ^1^H-NMR (CDCl_3_, minor-isomer) δ 1.43 (m, 2 H), 1.87 (dt, *J* = 11.5, 3.2 Hz, 2 H), 2.15–2.30 (m, 2 H), 2.03 (d, *J* = 7.3 Hz, 3 H), 3.51–3.62 (m, 4 H), 3.61 (br t, *J* = 3.2 Hz, 1 H), 3.74 (s, 3 H), 3.93–4.01 (m, 1 H), 5.32 (dq, *J* = 14.6, 7.3 Hz, 1 H), 5.46 (dt, *J* = 14.6, 6.6 Hz, 1 H), 6.70 (d, *J* = 8.8 Hz, 2 H), 6.77 (d, *J* = 8.8 Hz, 2 H); ^13^C-NMR (CDCl_3_, minor-isomer) δ 18.0, 32.5, 37.4, 52.7, 55.7, 63.9, 71.1, 74.3, 114.9, 117.0, 125.2, 130.7, 140.8, 154.0.

*(2S, 3S, 5S, 7S)-5-(4-Methoxyphenylamino)-7-methyl-8-nonen-1,2,3-triol* (**1t**). (a mixture of major and minor isomers in a ratio of 2:1): IR (neat) 3267 (s), 2835 (s), 1639 (m), 1616 (m), 1417 (s), 1238 (s), 1180 (s), 1038 (s), 914 (s), 824 (s), 606 (s) cm^−1^; ^1^H-NMR (CDCl_3_, major-isomer) δ 1.00 (d, *J* = 6.6 Hz, 3 H), 1.46–1.64 (m, 4 H), 1.88 (ddd, *J* = 14.4, 8.5, 3.4 Hz, 1 H), 2.26–2.33 (br-d, *J* = 7.3 Hz, 1 H), 3.48–3.80 (m, 3 H), 3.75 (s, 3 H), 3.97 (ddd, *J* = 11.5, 5.6, 2.9 Hz, 1 H), 4.89 (dd, *J* = 17.2, 1.0 Hz, 1 H), 4.91 (dd, *J* = 9.7, 1.7 Hz, 1 H), 5.65 (ddd, *J* = 17.2, 9.7, 7.3 Hz, 1 H), 6.66 (d, *J* = 9.0 Hz, 2 H), 6.77 (d, *J* = 9.0 Hz, 2 H); ^13^C-NMR (CDCl_3_, major-isomer) δ 20.7, 35.1, 37.4, 42.6, 51.2, 55.8, 63.9, 71.3, 74.0, 113.5, 114.9, 116.2, 141.0, 144.0, 153.0; ^1^H-NMR (CDCl_3_, minor-isomer) δ 0.94 (d, *J* = 6.8 Hz, 3 H), 1.46–1.64 (m, 4 H), 1.91 (ddd, *J* = 14.4, 8.5, 3.4 Hz, 1 H), 2.26–2.33 (br-d, *J* = 7.3 Hz, 1 H), 3.48–3.80 (m, 3 H), 3.75 (s, 3 H), 3.97 (ddd, *J* = 11.5, 5.6, 2.9 Hz, 1 H), 4.89 (dd, *J* = 17.2, 1.0 Hz, 1 H), 4.91 (dd, *J* = 9.7, 1.7 Hz, 1 H), 5.65 (ddd, *J* = 17.2, 9.7, 7.3 Hz, 1 H), 6.67 (d, *J* = 10.5 Hz, 2 H), 6.69 (d, *J* = 10.2 Hz, 2 H); ^13^C-NMR (CDCl_3_, minor-isomer) δ 20.7, 34.9, 37.2, 43.2, 51.6, 55.7, 63.8, 71.3, 74.2, 113.5, 114.9, 116.2, 141.0, 144.2, 153.0; HRMS, calcd for C_17_H_27_NO_4_: 309.1940. Found *m*/*z* (relative intensity): 310.1951 (M^+^+1, 19), 309.1932 (M^+^, 100), 248.1652 (12), 247.1566 (5).

*(2S, 3S, 5S, 7S)-5-(2-Methoxyphenylamino)-7-methyl-8-nonen-1,2,3-triol* (**1u**). (a mixture of major and minor isomers in a ratio of 2:1): IR (neat) 3379 (s), 3071 (s), 2932 (s), 2360 (s), 1596 (s), 1512 (s), 1458 (s), 1227 (s), 1026 (s), 910 (s), 733 (s), 679 (s) cm^−1^; ^1^H-NMR (CDCl_3_, major-isomer) δ 0.99 (d, *J* = 6.6 Hz, 3 H), 1.42–1.52 (br d, *J* = 6.6 Hz, 4 H), 2.22–2.27 (br d, *J* = 6.6 Hz, 1 H), 3.42–3.43 (m, 1 H), 3.57–3.58 (m, 2 H), 3.76–3.78 (m, 3 H), 3.77 (s, 3 H), 4.01 (br, 3 H), 4.83 (d, *J* = 17.3 Hz, 1 H), 4.86 (d, *J* = 10.0 Hz, 1 H), 5.62 (ddd, *J* = 17.3, 10.0, 7.5 Hz, 1 H), 6.56–6.64 (m, 1 H), 6.70–6.72 (m, 2 H), 6.78 (t, *J* = 7.6 Hz, 1 H); ^13^C-NMR (CDCl_3_, major-isomer) δ 20.5, 34.7, 38.4, 43.3, 48.4, 55.3, 63.1, 70.1, 74.5, 109.7, 110.8, 113.1, 116.2, 121.2, 137.6, 143.9, 146.6; ^1^H-NMR (CDCl_3_, minor-isomer) δ 0.95 (d, *J* = 6.3 Hz, 3 H), 1.65–1.78 (m, 4 H), 2.22–2.27 (br d, *J* = 6.6 Hz, 1 H), 3.42–3.43 (m, 1 H), 3.57–3.58 (m, 2 H), 3.76–3.78 (m, 3 H), 3.77 (s, 3 H), 4.01 (br, 3 H), 4.83 (d, *J* = 17.3 Hz, 1 H), 4.86 (d, *J* = 10.0 Hz, 1 H), 5.62 (ddd, *J* = 17.3, 10.0, 7.5 Hz, 1 H), 6.56–6.64 (m, 1 H), 6.70–6.72 (m, 2 H), 6.78 (t, *J* = 7.6 Hz, 1 H); ^13^C-NMR (CDCl_3_, minor-isomer) δ 20.7, 34.6, 38.4, 43.3, 48.2, 55.3, 63.1, 70.1, 74.5, 109.7, 110.8, 113.2, 116.4, 121.2, 137.5, 143.9, 146.6; HRMS, calcd for C_17_H_27_NO_4_: 309.1940. Found *m*/*z* (relative intensity): 309.1922 (M^+^, 100).

*(2S, 3S, 5S, 7S)-5-(3,4-Dimethoxyphenylamino)-7-methyl-8-nonen-1,2,3-triol* (**1v**). (a mixture of major and minor isomers in a ratio of 2:1):IR (neat) 3379 (s), 3078 (m), 2932 (s), 1705 (m), 1612 (s), 1458 (s), 1234 (s), 1026 (s), 918 (m), 733 (s) cm^−1^; ^1^H-NMR (CDCl_3_, major-isomer) δ 1.00 (d, *J* = 6.6 Hz, 3 H), 1.45–1.63 (m, 4 H), 1.86 (ddd, *J* = 14.2, 9.3, 3.2 Hz, 1 H), 2.20–2.40 (m, 1 H), 3.12 (br, 1 H), 3.56 (br dd, *J* = 9.5, 5.4 Hz, 1 H), 3.75 (m, 2 H), 3.80 (s, 3 H), 3.82 (s, 3 H), 3.95 (ddd, *J* = 8.8, 5.4, 3.1 Hz, 1 H), 4.90 (d, *J* = 17.1 Hz, 1 H), 4.92 (d, *J* = 10.5 Hz, 1 H), 5.65 (ddd, *J* = 17.1, 10.5, 7.9 Hz, 1 H), 6.23 (dd, *J* = 8.5, 2.4 Hz, 1 H), 6.32 (d, *J* = 2.4 Hz, 1 H), 6.72 (d, *J* = 8.5 Hz, 1 H); ^13^C-NMR (CDCl_3_, major-isomer) δ 20.7, 35.0, 37.7, 42.7, 50.8, 55.8, 56.6, 56.8, 63.6, 70.9, 100.5, 105.7, 113.4, 122.3, 132.3, 141.7, 144.0, 149.9; ^1^H-NMR (CDCl_3_, minor-isomer) δ 0.96 (d, *J* = 6.8 Hz, 3 H), 1.45–1.63 (m, 4 H), 1.95 (ddd, *J* = 14.4, 8.8, 3.2 Hz, 1 H), 2.20–2.40 (m, 1 H), 3.12 (br, 1 H), 3.56 (br dd, *J* = 9.5, 5.4 Hz, 1 H), 3.75 (m, 2 H), 3.80 (s, 3 H), 3.82 (s, 3 H), 4.00 (m, 1 H), 4.88 (d, *J* = 18.8 Hz, 1 H), 4.98 (br d, *J* = 10.8 Hz, 1 H), 5.58 (ddd, *J* = 18.8, 10.8, 8.0 Hz, 1 H), 6.29 (dt, *J* = 8.5, 2.4 Hz, 1 H), 6.34 (d, *J* = 2.4 Hz, 1 H), 6.74 (d, *J* = 8.5 Hz, 1 H); ^13^C-NMR (CDCl_3_, minor-isomer) δ 20.7, 35.0, 37.7, 42.7, 50.8, 55.8, 56.6, 56.8, 63.6, 70.9, 100.5, 105.7, 113.4, 122.3, 132.3, 141.7, 144.0, 149.9; HRMS, calcd for C_18_H_29_NO_5_: 339.2046. Found *m*/*z* (relative intensity): 340 (M^+^+1, 24), 339.2039 (M^+^, 100), 324 (7), 321 (3).

*(2S, 3S, 5S, 7S)-5-Phenylamino-7-methyl-8-nonen-1,2,3-triol* (**1w**). (a mixture of major and minor isomers in a ratio of 2:1): IR (neat) 3400 (s), 2870 (s), 1639 (s), 1602 (s), 1502 (s), 1259 (s), 1180 (s), 993 (s), 873 (s), 754 (s), 667 (s) cm^−1^; ^1^H-NMR (CDCl_3_, major-isomer) δ 1.01 (d, *J* = 6.8 Hz, 3 H), 1.52–1.58 (m, 4 H), 1.85 (ddd, *J* = 14.4, 9.3, 3.4 Hz, 2 H), 2.28–2.35 (br d, *J* = 7.2 Hz, 1 H), 3.55 (br d, *J* = 9.3 Hz, 1 H), 3.77 (m, 3 H), 3.95 (ddd, *J* = 9.3, 5.4, 2.7 Hz, 1 H), 4.88 (dt, *J* = 17.4, 1.0 Hz, 1 H), 4.92 (dt, *J* = 9.9, 0.9 Hz, 1 H), 5.65 (ddd, *J* = 17.4, 9.9, 7.5 Hz, 1 H), 6.65 (dd, *J* = 8.5, 1.0 Hz, 2 H), 6.70 (dd, *J* = 8.5, 7.3 Hz, 1 H), 7.16 (dd, *J* = 8.5, 7.3 Hz, 2 H); ^13^C-NMR (CDCl_3_, major-isomer) δ 20.9, 35.1, 38.1, 43.1, 49.5, 63.7, 71.0, 74.1, 113.9, 114.5, 129.3, 144.0, 147.5; ^1^H-NMR (CDCl_3_, minor-isomer) δ 0.97 (d, *J* = 6.8 Hz, 3 H), 1.52–1.58 (m, 4 H), 1.85 (ddd, *J* = 14.4, 9.3, 3.4 Hz, 2 H), 2.28–2.35 (br d, *J* = 7.2 Hz, 1 H), 3.55 (br d, *J* = 9.3 Hz, 1 H), 3.77 (m, 3 H), 3.95 (ddd, *J* = 9.3, 5.4, 2.7 Hz, 1 H), 4.88 (dt, *J* = 17.4, 1.0 Hz, 1 H), 4.92 (dt, *J* = 9.9, 0.9 Hz, 1 H), 5.59 (br dd, *J* = 9.6, 7.5 Hz, 1 H), 6.65 (dd, *J* = 8.5, 1.0 Hz, 2 H), 6.70 (dd, *J* = 7.6, 6.8 Hz, 1 H), 7.16 (dd, *J* = 8.5, 7.3 Hz, 2 H); ^13^C-NMR (CDCl_3_, minor-isomer) δ 20.9, 35.0, 38.1, 43.1, 49.5, 63.7, 71.0, 74.1, 113.6, 115.5, 129.3, 144.0, 147.5; HRMS, calcd for C_16_H_25_NO_3_: 279.1834. Found *m*/*z*(relative intensity): 280.1877 (M^+^+1, 19), 279.1831 (M^+^, 100), 278.1753 (4), 248.1628 (6), 217.1507 (3).

*(2S, 3S, 5S, 7S)-4-(4-Chlorophenylamino)-7-methyl-8-nonen-1,2,3-triol* (**1x**). (a mixture of major and minor isomers in a ratio of 2:1): IR (neat) 3364 (s), 3078 (s), 2924 (s), 2361 (s), 1705 (s), 1597 (s), 1504 (s), 1319 (s), 1258 (s), 1180 (s), 918 (s), 818 (s), 671 (s) cm^−1^; ^1^H-NMR (CDCl_3_, major-isomer) δ 0.91 (d, *J* = 6.6 Hz, 3 H), 1.38 (t, *J* = 6.6 Hz, 2 H), 1.18–1.60 (m, 2 H), 2.18 (br quint, d, *J* = 6.6 Hz, 1 H), 3.34–3.39 (m, 1 H), 3.49–3.57 (m, 3 H), 3.70–3.72 (m, 2 H), 3.78 (br, 3 H), 4.76 (d, *J* = 17.2 Hz, 1 H), 4.83 (d, *J* = 10.2 Hz, 1 H), 5.52 (ddd, *J* = 17.2, 10.2, 7.9 Hz, 1 H), 6.46 (d, *J* = 8.8 Hz, 2 H), 6.97 (d, *J* = 8.8 Hz, 2 H); ^13^C-NMR (CDCl_3_, major-isomer) δ 20.0, 34.9, 38.4, 43.1, 49.2, 63.1, 70.2, 74.5, 113.6, 114.5, 121.6, 129.0, 143.9, 146.5; ^1^H-NMR (CDCl_3_, minor-isomer) δ 0.88 (d, *J* = 6.8 Hz, 3 H), 1.38 (t, *J* = 6.6 Hz, 2 H), 1.18–1.60 (m, 2 H), 2.18 (br quint, d, *J* = 6.6 Hz, 1 H), 3.34–3.39 (m, 1 H), 3.49–3.57 (m, 3 H), 3.70–3.72 (m, 2 H), 3.78 (br, 3 H), 4.76 (d, *J* = 17.2 Hz, 1 H), 4.83 (d, *J* = 10.2 Hz, 1 H), 5.52 (ddd, *J* = 17.2, 10.2, 7.9 Hz, 1 H), 6.51 (d, *J* = 8.8 Hz, 2 H), 7.02 (d, *J* = 8.8 Hz, 2 H); ^13^C-NMR (CDCl_3_, minor-isomer) δ 21.0, 34.8, 38.4, 42.7, 49.2, 63.1, 70.2, 74.4, 113.7, 114.5, 121.6, 129.1, 143.8, 146.5; HRMS, calcd for C_16_H_24_ClNO_3_: 313.1445. Found *m*/*z* (relative intensity): 314.1446 (M^+^+1, 6), 313.1418 (M^+^, 29), 245.0748 (13), 244.0726 (100).

*(2S, 3S, 5S, 7S)-5-(4-Methoxyphenylamino)-7-vinyl-11-dodecen-1,2,3-triol* (**1y**). (a mixture of major and minor isomers in a ratio of 1:1): IR (neat) 3300 (m), 2912 (s), 2835 (s), 1639 (s), 1618 (s), 1500 (s), 1456 (s), 1294 (s), 1238 (s), 1180 (s), 1039 (s), 916 (s), 821 (s), 748 (s) cm^−1^; ^1^H-NMR (CDCl_3_, major-isomer) δ 1.18–1.36 (m, 2 H), 1.46–1.71 (m, 2 H), 1.56 (s, 3 H), 1.67 (s, 3 H), 1.82–2.00 (m, 2 H), 1.91 (ddd, *J* = 14.1, 9.2, 3.7 Hz, 2 H), 2.04–2.16 (m, 1 H), 3.53–3.59 (br dd, *J* = 9.2, 5.3 Hz, 1 H), 3.62–3.65 (br d, *J* = 3.7 Hz, 1 H), 3.71–3.85 (m, 2 H), 3.75 (s, 3 H), 3.95–4.00 (m, 1 H), 4.86 (dd, *J* = 17.1, 1.7 Hz, 1 H), 4.99 (dd, *J* = 10.1, 1.7 Hz, 1 H), 5.02–5.06 (t m, *J* = 7.1 Hz, 1 H), 5.49 (ddd, *J* = 17.1, 10.1, 7.1 Hz, 1 H), 6.67 (d, *J* = 8.9 Hz, 2 H), 6.76 (d, *J* = 8.9 Hz, 2 H); ^13^C-NMR (CDCl_3_, major-isomer) δ 17.8, 25.6, 25.8, 35.4, 37.6, 40.7, 41.1, 51.6, 55.8, 63.8, 71.4, 74.1, 114.9, 115.5, 116.5, 124.2, 131.5, 142.6, 142.8, 153.2; ^1^H-NMR (CDCl_3_, minor-isomer) δ 1.18–1.36 (m, 2 H), 1.46–1.71 (m, 2 H), 1.56 (s, 3 H), 1.67 (s, 3 H), 1.82–2.00 (m, 2 H), 1.91 (ddd, *J* = 14.1, 9.2, 3.7 Hz, 2 H), 2.04–2.16 (m, 1 H), 3.53–3.59 (br dd, *J* = 9.2, 5.3 Hz, 1 H), 3.62–3.65 (br d, *J* = 3.7 Hz, 1 H), 3.71–3.85 (m, 2 H), 3.75 (s, 3 H), 3.95–4.00 (m, 1 H), 4.80 (dd, *J* = 17.6, 1.6 Hz, 1 H), 4.97 (dd, *J* = 10.2, 1.6 Hz, 1 H), 5.02–5.06 (t m, *J* = 7.1 Hz, 1 H), 5.49 (ddd, *J* = 17.1, 10.1, 7.1 Hz, 1 H), 6.67 (d, *J* = 8.9 Hz, 2 H), 6.78 (d, *J* = 9.0 Hz, 2 H); ^13^C-NMR (CDCl_3_, minor-isomer) δ 17.8, 25.4, 25.7, 35.4, 37.6, 40.7, 41.1, 51.6, 55.7, 63.8, 71.4, 74.2, 114.8, 115.5, 116.5, 124.2, 131.5, 142.6, 142.8, 153.8; HRMS, calcd for C_22_H_35_NO_4_: 377.2566. Found *m*/*z* (relative intensity): 377.2561 (M^+^, 100).

General procedure for the Ni-catalyzed homoallylation of N,O-acetals prepared from carbohydrate and primary amines with dienes (entry 8, Table 4): A solution of D-ribose (150 mg, 1 mmol) and *p*-anisidine (246 mg, 2 mmol) in dry DMF (5 mL) was refluxed for 120 min under nitrogen. The solvent was removed by distillation under reduced pressure (azeotropic removal of water). A mixture of Ni(cod)_2_ (27.5 mg, 0.1 mmol) and isoprene (800 μL, 8 mmol) dissolved in THF (2 mL) and triethylborane (6.0 mmol, 1.0 M THF solution) were successively added to the flask containing *N*,*O*-acetal. The reaction mixture was stirred at 50 °C for 48 h, and the reaction mixture was diluted with 30 mL of EtOAc and washed with sat. NaHCO_3_, and brine. The organic phase was dried (MgSO_4_) and concentrated *in vacuo* to give a colorless oil, which was subjected to column chromatography over silica gel (hexane/EtOAc = 0/100 v/v) to give 2 (192 mg, 57%) in a 2:1 ratio. R_f_ = 0.30 (hexane/EtOAc = 0/100 v/v).

*5-[(4-Methoxyphenyl)amino]-7-methylnon-8-ene-1,2,3,4-tetraol* (**2**). (a mixture of major and minor isomers in a ratio of 2:1): IR (neat) 3400 (m), 2930 (m), 1653 (s), 1539 (s), 1456 (s), 1231 (s), 1180 (s), 1038 (s), 916 (s), 829 (s), 735 (s), 667 (s) cm^−1^; ^1^H-NMR (CDCl_3_, major-isomer) δ 0.98 (d, *J* = 6.6 Hz, 3 H), 1.48–1.57 (br d, *J* = 13.8 Hz, 2 H), 1.74 (ddd, *J* = 13.8, 10.4, 2.9 Hz, 1 H), 2.27–2.29 (br d, *J* = 7.7 Hz, 1 H), 3.61–3.69 (m, 2 H), 3.74 (s, 3 H), 3.76–3.90 (m, 3 H), 4.82 (d, *J* = 17.1 Hz, 1 H), 4.92 (dd, *J* = 10.2, 1.7 Hz, 1 H), 5.60 (ddd, *J* = 17.1, 10.2, 7.7 Hz, 1 H), 6.78 (d, *J* = 10.6 Hz, 2 H), 6.79 (d, *J* = 10.6 Hz, 2 H); ^13^C-NMR (CDCl_3_, major-isomer) δ 21.7, 35.2, 37.0, 55.9, 57.5, 63.6, 73.0, 73.4, 73.9, 114.4, 115.0, 117.4, 143.9, 153.9, 162.5; ^1^H-NMR (CDCl_3_, minor-isomer) δ 0.96 (d, *J* = 6.8 Hz, 3 H), 1.48–1.57 (br d, *J* = 13.8 Hz, 2 H), 1.74 (ddd, *J* = 13.8, 10.4, 2.9 Hz, 1 H), 2.27–2.29 (br d, *J* = 7.7 Hz, 1 H), 3.61–3.69 (m, 2 H), 3.75 (s, 3 H), 3.76–3.90 (m, 3 H), 4.82 (d, *J* = 17.1 Hz, 1 H), 4.91 (d, *J* = 11.2 Hz, 1 H), 5.60 (ddd, *J* = 17.1, 10.2, 7.7 Hz, 1 H), 6.78 (d, *J* = 10.6 Hz, 2 H), 6.79 (d, *J* = 10.6 Hz, 2 H); ^13^C-NMR (CDCl_3_, minor-isomer) δ 21.7, 34.9, 36.6, 55.9, 57.5, 63.6, 73.2, 73.7, 73.9, 114.4, 115.1, 117.8, 144.2, 153.9, 162.5; HRMS, calcd for C_17_H_27_NO_5_: 325.1889. Found *m*/*z* (relative intensity): 326.1940 (M^+^+1, 20), 325.1873 (M^+^, 100).

*(2R, 3S, 4R, 6S, 8S)-6-(4-Methoxyphenylamino)-8-methyl-9-decen-1,2,3,4-tetraol* (**3**). (a mixture of major and minor isomers in a ratio of 1:1): IR (KBr) 3285 (s), 2937 (m), 2924 (m), 1514 (s), 1412 (m), 1240 (s), 1074 (s), 1040 (s), 822 (w), 640 (w) cm^−1^; ^1^H-NMR (CDCl_3_, major-isomer) δ 1.00 (d, *J* = 6.8 Hz, 3 H), 1.44–1.62 (m, 4 H), 2.04–2.11 (ddd, *J* = 5.1, 9.3, 12.7, 1 H), 2.26 (br d, *J* = 7.5 Hz, 1 H), 3.51–3.83 (m, 5 H), 3.74 (s, 3 H), 4.16–4.20 (m, 1 H), 4.89 (d, *J* = 18.0 Hz, 1 H), 4.92 (d, *J* = 10.5 Hz, 1 H), 5.63 (ddd, *J* = 17.1, 10.2, 7.8 Hz, 1 H), 6.73 (d, *J* = 9.0 Hz, 2 H), 6.77 (d, *J* = 9.0 Hz, 2 H); ^13^C-NMR (CDCl_3_, major-isomer) δ 20.8, 35.2, 37.9, 42.5, 51.7, 55.9, 64.0, 68.9, 72.9, 74.7, 113.7, 115.0, 116.7, 140.8, 144.1, 153.3; ^1^H-NMR (CDCl_3_, minor-isomer) δ 0.93 (d, *J* = 6.8 Hz, 3 H), 1.44–1.62 (m, 4 H), 2.04–2.11 (ddd, *J* = 5.1, 9.3, 12.7, 1 H), 2.26 (br d, *J* = 7.5 Hz, 1 H), 3.51–3.83 (m, 5 H), 3.76 (s, 3 H), 4.16–4.20 (m, 1 H), 4.88 (d, *J* = 17.3 Hz, 1 H), 4.97 (d, *J* = 9.3 Hz, 1 H), 5.63 (ddd, *J* = 17.1, 10.2, 7.8 Hz, 1 H), 6.72 (d, *J* = 10.7 Hz, 2 H), 6.79 (d, *J* = 9.0 Hz, 2 H); ^13^C-NMR (CDCl_3_, minor-isomer) δ 20.6, 35.0, 37.6, 42.9, 51.7, 55.5, 64.2, 68.9, 73.0, 74.3, 113.5, 115.0, 116.7, 140.8, 144.3, 153.3; HRMS, calcd for C_18_H_29_NO_5_: 339.2046. Found *m*/*z* (relative intensity): 340.2072 (M^+^+1, 20), 339.2037 (M^+^, 100).

## 4. Conclusions

Homoallylation of *N*,*O*-acetals prepared from cyclic hemiacetals and primary amines provided hydroxyhomoallylamines in the presence of a Ni-catalyst and triethylborane. *N*,*O*-Acetals prepared from glycolaldehyde dimer and glyceraldehyde dimer with primary amines underwent homoallylation with conjugated dienes to give 2-amino-5-hexenols. Under similar conditions, *N*,*O*-acetals from carbohydrates with primary amines provided polyhydroxybishomoallylamines in good to reasonable yields. These reactions can be applied to the efficient synthesis of physiologically active molecules, such as polyhydroxyamines, terpenes, and neurotransmitters, from non-protected carbohydrates.

## Supplementary Materials

Supplementary materials can be accessed at: : http://www.mdpi.com/1420-3049/19/7/9288/s1.

## References

[1] Tamaru Y. (2005). Modern Organonickel Chemistry.

[2] Behr A., Neubert P. (2014). Piperylene—A versatile basic chemical in catalysis. ChemCatChem.

[3] Bertelo C., Schwartz J., Hydrozirconation V. (1976). γ,δ-Unsaturated aldehydes and halides from 1,3-dienes via organozirconium (IV) intermediates. J. Am. Chem. Soc..

[4] Yasuda H., Tatsumi K., Nakamura A. (1985). Unique chemical behavior and bonding of early-transition-metal-diene complexes. Acc. Chem. Res..

[5] Kimura M., Ezoe A., Shibata K., Tamaru Y. (1998). Novel and highly regio- and stereoselective nickel-catalyzed homoallylation of benzaldehyde with 1,3-dienes. J. Am. Chem. Soc..

[6] Kimura M., Ezoe A., Mori M., Iwata K., Tamaru Y. (2006). Regio- and stereoselective nickel-catalyzed homoallylation of aldehydes with 1,3-dienes. J. Am. Chem. Soc..

[7] Kimura M., Fujimatsu H., Ezoe A., Shibata K., Shimizu M., Matsumoto S., Tamaru Y. (1999). Nickel-catalyzed homoallylation of aldehydes and ketones with 1,3-dienes and complementary promotion by diethylzinc or triethylborane. Angew. Chem. Int. Ed..

[8] Kimura M., Ezoe A., Tanaka S., Tamaru Y. (2001). Nickel-catalyzed homoallylation of aldehydes in the presence of water and alcohols. Angew. Chem. Int. Ed..

[9] Kimura M., Miyachi A., Kojima K., Tanaka S., Tamaru Y. (2004). Highly stereo- and regioselective Ni-catalyzed homoallylation of aldimines with conjugated dienes promoted by diethylzinc. J. Am. Chem. Soc..

[10] Kim H.-J., Ricardo A., Illangkoon H.-I., Kim M.J., Carrigan M.A., Frye F., Benner S.A.  (2011). Synthesis of carbohydrates in mineral-guided prebiotic cycles. J. Am. Chem. Soc..

[11] Archer R.M., Royer S.F., Mahy W., Winn C.L., Danson M.J., Bull S.D. (2013). Syntheses of 2-keto-3-deoxy-d-xylonate and 2-keto-3-deoxy-l-arabinonate as stereochemical probes for demonstrating the metabolic promiscuity of sulfolobus solfataricus towards d-xylose and l-arabinose. Chem. Eur. J..

[12] Li Z., Cai L., Wei M., Wang P.G. (2012). One-pot four-enzyme synthesis of ketones with fructose 1,6-bisphosphate aldolases from staphylococcus carnosus and rabit muscle. Carbohydr. Res..

[13] Kodato S., Nakagawa M., Nakayama K., Hino T. (1989). Synthesis of cerebroside B_1b_ with antiulcerogenic activity I. Synthesis of ceramides with optically active α-hydroxypalmitic acids. Tetrahedron.

[14] Mukai R., Horino Y., Tanaka S., Tamaru Y., Kimura M. (2004). Pd(0)-catalyzed amphiphilic activation of bis-allyl alcohol and ether. J. Am. Chem. Soc..

[15] Kimura M., Shimizu M., Tanaka S., Tamaru Y. (2005). Pd-catalyzed nucleophilic allylic alkylation of aliphatic aldehydes by the use of allyl alcohols. Tetrahedron.

[16] Yamaguchi Y., Hasimoto M., Tohyama K., Kimura M. (2011). Nucleophilic allylation of *N*,*O*-acetals with allylic alcohols promoted by Pd/Et_3_B and Pd/Et_2_Zn systems. Tetrahedron Lett..

