# Supplementary material for: Ni-Catalyzed Homoallylation of Polyhydroxy N,O-Acetals with Conjugated Dienes Promoted by Triethylborane
Source: Molecules. 2014 Jul 2;19(7):9288–306. doi: 10.3390/molecules19079288 (PMC6271873; doi:10.3390/molecules19079288)
Supplement: Supplementary file 1 [file molecules-19-09288-s001.pdf]

Supporting Information

General Procedures

Distillation were carried out in a Kugelrohr apparatus (GTO-350RG glass tube oven, SIBATA, Soka (Saitama, Japan). Boiling points are meant to refer to the oven temperature (±1 °C). Microanalyses were performed by the Instrumental Analysis Center of Nagasaki University. Analysis agreed with the calculated values within ±0.4%. High resolution mass spectra (HRMS) were measured with a JEOL JMSDX303 instrument (JEOL, Akishima, Tokyo, Japan). Infrared spectra were recorded with a JASCO A-100 (Hachioji, Tokyo, Japan) or Shimadzu FTIR-8700 (Kyoto, Japan) infrared spectrophotometer. ^{1}H (400 MHz) and ^{13}C-NMR spectra (100 MHz) were measured on JEOL-GX400 instrument with tetramethylsilane as an internal standard. Chemical shift values were given in ppm downfield from the internal standard.

Tetrahydrofuran, toluene, and diethyl ether were dried and distilled from benzophenone-sodium immediately prior to use under nitrogen atmosphere. DMF was distilled over calcium chloride. Triethylborane (1 M THF, KANTO Kagaku, Tokyo, Japan), $Ni(cod)_2$ (KANTO Kagaku, Tokyo, Japan) were used without further purification. Isoprene, myrcene, glycolaldehyde dimmer, glyceraldehyde dimer, 2-deoxy-D-ribose, D-ribose, 2-deoxy-D-glucose, aniline, *p*-methoxyaniline, *o*-methoxyaniline, *p*-bromoaniline, benzylamine were purchased and used without purification. 1,3-Butadiene was purchased (Tokyo Kasei Kogyo Co., Ltd., Tokyo, Japan), and was liquefied by cooling at −78 °C (dry ice/isopropanol) prior to use under argon atmosphere. 1,3-Butadiene could be measured by syringe kept cool in the freezer well beforehand, and then was introduced into the reaction mixture at room temperature. Tetrahydrofuran-2-ol, tetrahydro-2*H*-pyran-2-ol, oxepane-2-ol, 5-(naphthalen-2-yl) tetrahydrofuran-2-ol, and all of the substrates in Table 1 were prepared according to the literature [1–4].

Typical Procedure for Ni-Catalyzed Homoallylation of N,O-Acetal with Isoprene (Entry 4, Table 1)

A solution of tetrahydro-2*H*-pyran-2-ol (102 mg, 1 mmol) and *p*-anisidine (246 mg, 2 mmol) in dry THF (2 mL) was stirred overnight under nitrogen. A mixture of $Ni(cod)_2$ (27.5 mg, 0.1 mmol) and isoprene (400 μL, 4 mmol) dissolved in THF (2 mL) and triethylborane (3.6 mmol, 1.0 M THF solution) were successively added to the *N,O*-acetal solution. The reaction mixture was stirred at room temperature for 24 h. The reaction mixture was diluted with 30 mL of EtOAc and washed with sat. $NaHCO_3$, and brine. The organic phase was dried ($MgSO_4$) and concentrated in vacuo to give a colorless oil, which was subjected to column chromatography over silica gel (hexane/EtOAc = 2/1 v/v) to give **1d** (258 mg, 91%) in a 6:1 ratio. R_f = 0.30 (hexane/EtOAc = 4/1 v/v).

(4S,6S)-4-(4-Methoxyphenylamino)-6-methyloct-7-en-1-ol (**1a**). (a mixture of major and minor isomers in a ratio of 5:1): IR (neat) 3310 (s), 3071 (s), 2924 (s), 1643 (m), 1458 (s), 1065 (s), 910 (s), 741 (s) cm^{-1}; ^{1}H-NMR ($CDCl_3$, major-isomer) δ 0.99 (d, *J* = 6.7 Hz, 3 H), 1.60–1.69 (m, 6 H), 2.35 (qm, *J* = 6.7 Hz, 1 H), 2.52 (br, 1H), 3.32 (m, 1 H), 3.60 (t, *J* = 6.1 Hz, 2 H), 3.73 (s, 3 H), 4.92 (dm, *J* = 10.7 Hz, 1 H), 4.93 (dd, *J* = 16.9, 1.0 Hz, 1 H), 5.65 (ddd, *J* = 16.9, 10.7, 8.0 Hz, 1 H), 6.54 (dd, *J* = 6.6, 2.2 Hz, 2 H), 6.75 (m, 2 H); ^{13}C-NMR ($CDCl_3$, major-isomer) δ 21.0, 29.3, 32.0, 35.0, 42.5, 54.0, 55.8, 62.9, 63.0, 113.2, 114.9, 115.0, 115.4, 141.5, 141.8, 144.3; ^{1}H-NMR ($CDCl_3$, minor-isomer) δ 0.99 (d, *J* = 6.7 Hz, 3 H), 1.60–1.69 (m, 6 H), 2.35 (qm, *J* = 6.7 Hz, 1 H), 2.52 (br, 1H), 3.32 (m, 1 H),

3.60 (t, J = 6.1 Hz, 2 H), 3.73 (s, 3 H), 4.93 (dd, J = 16.9, 1.0 Hz, 1 H), 4.98 (dm, J = 17.1 Hz, 1 H), 5.65 (ddd, J = 16.9, 10.7, 8.0 Hz, 1 H), 6.54 (dd, J = 6.6, 2.2 Hz, 2 H), 6.75 (m, 2 H); ^{13}C-NMR ($CDCl_3$, minor-isomer) δ 20.8, 29.2, 32.0, 35.0, 45.3, 54.0, 55.8, 62.9, 63.0, 113.2, 114.8, 115.0, 115.4, 141.6, 141.8, 144.1; HRMS, calcd for $C_{16}H_{25}NO_2$: 263.1885. Found *m/z* (relative intensity): 264.1882 (M^++1, 18), 263.1850 (M^+, 97), 205.1411 (15), 204.1394 (100).

(4S,6S)-4-(4-Methoxyphenylamino)-6-methyl-1-(naphthalenyl) octen-7-ol (**1b**). (a mixture of major and minor isomers in a ratio of 2:1): IR (neat) 3366 (s), 2930 (s), 2359 (m), 1506 (s), 1238 (s), 1040 (s), 820 (s), 750 (s) cm^{-1}; ^{1}H-NMR ($CDCl_3$, major-isomer) δ 0.95 (d, J = 6.8 Hz, 3 H), 1.38–1.94 (m, 6 H), 2.29 (qm, J = 6.8 Hz, 1 H), 3.11 (br, 1 H), 3.32 (br q, J = 5.9 Hz, 1 H), 3.71 (s, 3 H), 4.78–4.82 (m, 1 H), 4.88 (dd, J = 18.3, 1.9 Hz, 1 H), 4.89 (dd, J = 10.8, 1.9 Hz, 1 H), 5.59 (ddd, J = 18.3, 10.8, 8.3 Hz, 1 H), 6.53 (d, J = 8.9 Hz, 2 H), 6.70 (dd, J = 8.9, 1.0 Hz, 2 H), 7.39–7.82 (m, 7 H); ^{13}C-NMR ($CDCl_3$, major-isomer) δ 20.9, 31.7, 35.0, 35.5, 42.3, 53.0, 55.7, 74.5, 113.2, 114.8, 115.3, 123.9, 124.4, 125.6, 125.7, 125.9, 126.0, 127.5, 128.0, 132.8, 133.1, 141.9, 144.1, 152.2; ^{1}H-NMR ($CDCl_3$, minor-isomer) δ 0.92 (d, J = 6.8 Hz, 3 H), 1.38–1.94 (m, 6 H), 2.29 (qm, J = 6.8 Hz, 1 H), 3.11 (br, 1 H), 3.32 (br q, J = 5.9 Hz, 1 H), 3.72 (s, 3 H), 4.83 (dm, J = 18.3 Hz, 1 H), 4.88 (dd, J = 18.3, 1.9 Hz, 1 H), 4.89 (dd, J = 10.8, 1.9 Hz, 1 H), 5.58–5.66 (m, 1 H), 6.53 (d, J = 8.9 Hz, 2 H), 6.70 (dd, J = 8.9, 1.0 Hz, 2 H), 7.39–7.82 (m, 7 H); ^{13}C-NMR (minor-isomer) δ 20.9, 31.2, 34.9, 35.7, 42.3, 52.7, 55.7, 74.4, 113.2, 114.8, 115.2, 124.0, 124.4, 125.6, 125.7, 125.9, 126.0, 127.5, 128.1, 132.6, 133.1, 141.3, 144.1, 152.1; HRMS, calcd for $C_{26}H_{31}NO_2$: 389.2355. Found *m/z* (relative intensity): 389.2340 (M^+, 100).

10-(4-Methoxyphenylamino)-7,12-dimethyl-13-tetradecen-7-ol (**1c**). (a mixture of major and minor isomers in a ratio of 3:1): IR (neat) 3379 (s), 2932 (s), 2359 (m), 1639 (s), 1514 (s), 1238 (s), 1043 (s), 912 (s), 818 (s) cm^{-1}; ^{1}H-NMR ($CDCl_3$, major-isomer) δ 0.88 (t, J = 6.4 Hz, 3 H), 0.96 (d, J = 6.8 Hz, 3 H), 1.12 –1.26 (m, 8 H), 1.42 (br, 3 H), 1.49–1.69 (m, 8 H), 2.29 (qm, J = 6.8 Hz, 1 H), 3.25–3.33 (m, 1 H), 3.74 (s, 3 H), 4.91 (dd, J = 10.7, 1.5 Hz, 1 H), 4.92 (dd, J = 17.5, 1.5 Hz, 1 H), 5.63 (ddd, J = 17.5, 10.7, 8.0 Hz, 1 H), 6.69–6.72 (m, 2 H), 6.75 (d, J = 9.3 Hz, 2 H); ^{13}C-NMR ($CDCl_3$, major-isomer) δ 14.1, 20.7, 20.9, 22.6, 26.9, 27.6, 29.8, 31.8, 35.0, 37.4, 37.6, 41.9, 55.6, 55.7, 72.6, 113.3, 114.0, 116.8, 143.4, 143.9, 144.0; ^{1}H-NMR ($CDCl_3$, minor-isomer) δ 0.88 (t, J = 6.4 Hz, 3 H), 0.97 (d, J = 6.8 Hz, 3 H), 1.12 –1.26 (m, 8 H), 1.42 (br, 3 H), 1.49–1.69 (m, 8 H), 2.29 (qm, J = 6.8 Hz, 1 H), 3.25–3.33 (m, 1 H), 3.74 (s, 3 H), 4.91 (dd, J = 10.7, 1.5 Hz, 1 H), 4.92 (dd, J = 17.5, 1.5 Hz, 1 H), 5.63 (ddd, J = 17.5, 10.7, 8.0 Hz, 1 H), 6.69–6.72 (m, 2 H), 6.75 (d, J = 9.0 Hz, 2 H); ^{13}C-NMR ($CDCl_3$, minor-isomer) δ 14.1, 20.8, 20.9, 22.6, 26.8, 27.6, 29.8, 31.8, 35.0, 37.4, 37.6, 41.9, 55.6, 55.7, 72.7, 113.4, 114.0, 116.7, 143.5, 143.9, 144.0; HRMS, calcd for $C_{23}H_{39}NO_2$: 361.2981. Found *m/z* (relative intensity): 361.2969 (M^+, 100).

5-[(4-Methoxyphenyl)amino]-7-methylnon-8-en-1-ol (**1d**) (a mixture of major and minor isomers in a ratio of 6:1): IR (neat) 3368 (s), 2934 (s), 1514 (m), 1458 (s), 1236 (s), 1040 (s), 820 (s) cm^{-1}; ^{1}H-NMR ($CDCl_3$, major-isomer) δ 1.00 (d, J = 6.6 Hz, 3 H), 1.37–1.58, (m, 8 H), 2.33 – 2.38 (m, 1 H), 3.31 (m, 1 H), 3.62 (t, J = 6.3 Hz, 2 H), 3.73 (s, 3 H), 4.92 (dd, J = 10.4, 1.1 Hz, 1 H), 4.93 (dd, J = 17.0, 1.1 Hz, 1 H), 5.65 (ddd, J = 17.0, 10.4, 8.1 Hz, 1 H), 6.52 (d, J = 8.9 Hz, 2 H), 6.74 (d, J = 8.9 Hz, 2 H); ^{13}C-NMR ($CDCl_3$, major-isomer) δ 21.1, 22.0, 32.8, 35.0, 35.2, 42.5, 52.1, 55.8, 62.8,

113.2, 114.4, 114.9, 142.1, 144.3, 151.6; ^{1}H-NMR ($CDCl_3$, minor-isomer) δ 0.98 (d, *J* = 6.8 Hz, 3 H), 1.37–1.58, (m, 8 H), 2.33–2.38 (m, 1 H), 3.31 (m, 1 H), 3.61 (t, *J* = 6.5 Hz, 2 H), 3.74 (s, 3 H), 4.92 (dd, *J* = 10.4, 1.1 Hz, 1 H), 4.93 (dd, *J* = 17.0, 1.1 Hz, 1 H), 5.65 (ddd, *J* = 17.0, 10.4, 8.1 Hz, 1 H), 6.52 (d, *J* = 8.9 Hz, 2 H), 6.74 (d, *J* = 8.9 Hz, 2 H); ^{13}C-NMR ($CDCl_3$, minor-isomer) δ 21.1, 21.9, 32.8, 35.0, 35.2, 42.1, 52.1, 55.8, 62.8, 113.2, 114.2, 114.8, 142.1, 144.3, 151.6; HRMS, calcd for $C_{17}H_{27}NO_2$: 277.2018. Found *m/z* (relative intensity): 278.2001 (M^++1, 1), 277.2014 (M^+, 2), 260.1983 (13), 204.1406 (100).

(5S,7S)-5-(4-Methoxyphenylamino)-3,3,7-trimethylnonen-8-ol (**1e**). (a mixture of major and minor isomers in a ratio of 4:1): IR (neat) 3373 (s), 2932 (s), 2359 (m), 1732 (s), 1514 (s), 1234 (s), 1042 (s), 818 (s) cm^{-1}; ^{1}H-NMR ($CDCl_3$, major-isomer) δ 0.94 (s, 6 H), 0.97 (d, *J* = 6.8 Hz, 3 H), 1.21–1.70(m, 6 H), 2.28 (qm, *J* = 6.8 Hz, 1 H), 3.02 (br, 1 H),3.34 (m, 1 H), 3.69 (s, 3 H), 3.66–3.76 (m, 2 H), 4.98 (dd, *J* = 17.7, 1.8 Hz, 1 H), 4.99 (dd, *J* = 10.0, 1.8 Hz, 1 H), 5.67 (ddd, *J* = 17.7, 10.0, 8.3 Hz, 1 H), 6.55 (br, 2 H), 6.75 (br d, *J* = 8.3 Hz, 2 H); ^{13}C-NMR ($CDCl_3$, major-isomer) δ 20.1, 21.6, 28.4, 28.5, 32.7, 44.5, 47.5, 47.9, 49.3, 55.0, 59.6, 113.6, 114.9, 115.0, 144.1, 144.7, 152.0; ^{1}H-NMR ($CDCl_3$, minor-isomer) δ 0.95 (s, 6 H), 0.99 (d, *J* = 6.6 Hz, 3 H), 1.21–1.70 (m, 6 H), 2.28 (qm, *J* = 6.8 Hz, 1 H), 3.02 (br, 1 H),3.34 (m, 1 H), 3.67 (s, 3 H), 3.66–3.76 (m, 2 H), 4.92 (dd, *J* = 10.2, 0.8 Hz, 1 H), 4.95 (dd, *J* = 16.9, 0.8 Hz, 1 H), 5.67 (ddd, *J* = 17.7, 10.0, 8.3 Hz, 1 H), 6.55 (br, 2 H), 6.75 (br d, *J* = 8.3 Hz, 2 H); ^{13}C-NMR ($CDCl_3$, minor-isomer) δ 20.1, 21.6, 28.4, 28.5, 32.6, 44.3, 47.5, 47.9, 49.3, 55.0, 59.6, 113.6, 114.8, 115.0, 144.1, 144.7, 152.0; HRMS, calcd for $C_{19}H_{31}NO_2$: 305.2355. Found *m/z* (relative intensity): 306.2373 (M^++1, 9), 305.2337 (M^+, 44), 237.1655 (18), 236.1608 (100), 235.1535 (19).

2-((3S,5S)-3-(4-Methoxyphenylamino)-5-methyl-6-heptenyl)phenol (**1f**). (a mixture of major and minor isomers in a ratio of 7:1): IR (neat) 3308 (s), 2930 (s), 1583 (m), 1506 (s), 1236 (s), 1040 (s), 822 (s), 754 (s) cm^{-1}; ^{1}H-NMR ($CDCl_3$, major-isomer) δ 0.84 (d, *J* = 6.8 Hz, 3 H), 1.11–1.68 (m, 4 H), 2.20 (qm, *J* = 6.8 Hz, 1 H), 2.65–2.71 (m, 2 H), 2.88–2.95 (m, 1 H), 3.13–3.29 (m, 1 H), 3.75 (s, 3 H), 4.86 (dd, *J* = 10.8, 0.8 Hz, 1 H), 4.87 (dd, *J* = 16.7, 0.8 Hz, 1 H), 5.57 (ddd, *J* = 16.7, 10.8, 7.8 Hz, 1 H), 6.78 (d, *J* = 8.6 Hz, 2 H), 6.84 (d, *J* = 8.6 Hz, 2 H), 6.73–6.92 (m, 2 H), 7.06–7.12 (m, 2 H); ^{13}C-NMR ($CDCl_3$, major-isomer) δ 20.2, 26.2, 34.8, 35.3, 40.0, 53.3, 55.6, 55.7, 112.9, 114.8, 116.3, 120.3, 127.2, 127.3, 127.4, 129.9, 130.0, 144.3, 154.8; ^{1}H-NMR ($CDCl_3$, minor-isomer) δ 0.89 (d, *J* = 6.3 Hz, 3 H), 1.11–1.68 (m, 4 H), 2.20 (qm, *J* = 6.8 Hz, 1 H), 2.65–2.71 (m, 2 H), 2.88–2.95 (m, 1 H), 3.13–3.29 (m, 1 H), 3.75 (s, 3 H), 4.86 (dd, *J* = 10.8, 0.8 Hz, 1 H), 4.87 (dd, *J* = 16.7, 0.8 Hz, 1 H), 5.57 (ddd, *J* = 16.7, 10.8, 7.8 Hz, 1 H), 6.78 (d, *J* = 8.6 Hz, 2 H), 6.84 (d, *J* = 8.6 Hz, 2 H), 6.73–6.92 (m, 2 H), 7.06–7.12 (m, 2 H); ^{13}C-NMR ($CDCl_3$, minor-isomer) δ 20.2, 26.2, 34.8, 35.3, 40.0, 53.3, 55.6, 55.7, 112.9, 114.6, 116.3, 120.6, 127.2, 127.3, 127.4, 129.9, 130.1, 144.3, 154.8; HRMS, calcd for $C_{21}H_{27}NO_2$: 325.2042. Found *m/z* (relative intensity): 326.2086 (M^++1, 18), 325.2045 (M^+, 78), 257.1349 (18), 256.1329 (100).

Tert-Butyl(5S,7S)-5-(4-methoxyphenylamino)-7-methylnon-8-enylcarbamate (**1g**). (a mixture of major and minor isomers in a ratio of 3:1):IR (neat) 2864 (s), 2359 (m), 1682 (s), 1539 (s), 1251 (s), 1173 (s), 910 (s), 750 (s) cm^{-1}; ^{1}H-NMR ($CDCl_3$, major-isomer) δ 1.01 (d, *J* = 6.8 Hz, 3 H), 1.34–1.51 (m, 8 H), 1.44 (s, 9 H), 2.32 (dm, *J* = 7.4 Hz, 1 H), 3.11 (br, 2 H), 3.67 (br, 1 H), 4.93 (dd, *J* = 10.2, 1.1 Hz, 1 H),

5.01 (dd, J = 17.4, 1.1 Hz, 1 H), 5.77 (ddd, J = 17.4, 10.2, 7.4 Hz, 1 H); ^{13}C-NMR ($CDCl_3$, major-isomer) δ 20.2, 22.6, 28.4, 30.1, 35.4, 37.2, 40.4, 44.5, 70.1, 79.0, 112.6, 114.9, 155.9; ^{1}H-NMR ($CDCl_3$, minor-isomer) δ 0.98 (d, J = 6.6 Hz, 3 H), 1.34–1.51 (m, 8 H), 1.44 (s, 9 H), 2.32 (dm, J = 7.4 Hz, 1 H), 3.11 (br, 2 H), 3.67 (br, 1 H), 4.88 (dm, J = 11.2 Hz, 1 H), 5.01 (dd, J = 17.4, 1.1 Hz, 1 H), 5.77 (ddd, J = 17.4, 10.2, 7.4 Hz, 1 H); ^{13}C-NMR ($CDCl_3$, minor-isomer) δ 19.8, 22.6, 28.4, 30.0, 35.4, 37.2, 40.4, 44.3, 69.8, 79.0, 112.0, 114.6, 155.9; HRMS, calcd for $C_{15}H_{29}NO_3$: 271.2147. Found *m/z* (relative intensity): 376.2731 (M^+, 100).

(6S,8S)-6-(4-Methoxyphenylamino)-8-methyldec-9-en-1-ol (**1h**). (a mixture of major and minor isomers in a ratio of 4:1): IR (neat) 3364 (s), 2934 (s), 1614 (m), 1514 (s), 1238 (s), 1038 (s), 822 (s) cm^{-1}; ^{1}H-NMR ($CDCl_3$, major-isomer) δ 1.00 (d, J = 6.8 Hz, 3 H), 1.21–1.58, (m, 10 H), 2.34–2.38 (m, 1 H), 3.27–3.32 (m, 1 H), 3.61 (t, J = 6.6 Hz, 2 H), 3.74 (s, 3 H), 4.92 (dd, J = 10.2, 0.9 Hz, 1 H), 4.93 (dd, J = 17.0, 0.9 Hz, 1 H), 5.65 (ddd, J = 17.0, 10.2, 8.1 Hz, 1 H), 6.63 (d, J = 9.1 Hz, 2 H), 6.74 (d, J = 9.1 Hz, 2 H); ^{13}C-NMR ($CDCl_3$, major-isomer) δ 21.2, 25.6, 25.9, 32.8, 35.1, 35.3, 42.4, 55.8, 55.9, 62.9, 113.3, 114.9, 116.4, 139.8, 144.3, 152.8; ^{1}H-NMR ($CDCl_3$, minor-isomer) δ 0.97 (d, J = 6.8 Hz, 3 H), 1.21–1.58, (m, 10 H), 2.34–2.38 (m, 1 H), 3.27–3.32 (m, 1 H), 3.61 (t, J = 6.4 Hz, 2 H), 3.74 (s, 3 H), 4.92 (dd, J = 10.2, 0.9 Hz, 1 H), 4.93 (dd, J = 17.0, 0.9 Hz, 1 H), 5.65 (ddd, J = 17.0, 10.2, 8.1 Hz, 1 H), 6.63 (d, J = 9.1 Hz, 2 H), 6.74 (d, J = 9.1 Hz, 2 H); ^{13}C-NMR ($CDCl_3$, minor-isomer) δ 21.2, 25.6, 25.9, 32.8, 35.1, 35.3, 42.4, 55.8, 55.9, 63.1, 113.3, 114.8, 116.4, 139.8, 144.3, 152.8; HRMS, calcd for $C_{18}H_{29}NO_2$: 291.2196. Found *m/z* (relative intensity): 292.2236 (M^++1, 15), 291.2196 (M^+, 65), 223.1501 (13), 222.1497 (100).

2-(4-Methoxyphenylamino)-5-hexenol (**1i**). IR (neat) 3375 (s), 3076 (m), 2936 (s), 1639 (s), 1514 (s), 1464 (s), 1238 (s), 1038 (s), 822 (s) cm^{-1}; ^{1}H-NMR ($CDCl_3$) δ 1.42 (quin, J = 7.5 Hz, 2 H), 2.14 (br q, J = 6.8 Hz, 1 H), 2.25 (br q, J = 6.4 Hz, 1 H), 2.69 (br, 1 H), 3.40 (m, 2 H), 3.49 (dd, J = 10.9, 6.1 Hz, 1 H), 3.51 (dd, J = 10.9, 6.1 Hz, 1 H), 3.74 (s, 3 H), 4.94 (dm, J = 9.7 Hz, 1 H), 5.00 (dt, J = 16.1,1.9 Hz, 1 H), 5.78 (ddt, J = 16.1, 9.7, 6.4 Hz, 1 H), 6.64 (dd, J = 6.6, 2.4 Hz, 2 H), 6.74 (dd, J = 6.6, 2.4 Hz, 2 H); ^{13}C-NMR ($CDCl_3$) δ 28.7, 30.4, 55.8, 56.3, 64.1, 114.8, 115.7, 116.4, 137.8, 139.8, 152.7; HRMS, calcd for $C_{13}H_{19}NO_2$: 221.1416. Found *m/z* (relative intensity): 222.1449 (M^++1, 4), 221.1401 (M^+, 28), 191.1235 (14), 190.1195 (100).

(E)-2-(4-Methoxyphenylamino)-4-hexenol (**1i'**). ^{1}H-NMR ($CDCl_3$) δ 1.64 (dm, J = 7.6 Hz, 3 H), 2.02 (m, 2 H), 3.40 (m, 2 H), 3.49 (dd, J = 10.9, 6.1 Hz, 1 H), 3.51 (dd, J = 10.9, 6.1 Hz, 1 H), 3.74 (s, 3 H), 5.40 (m, 1 H), 5.50 (m, 1 H), 6.64 (dd, J = 6.6, 2.4 Hz, 2 H), 6.74 (dd, J = 6.6, 2.4 Hz, 2 H); ^{13}C-NMR ($CDCl_3$) δ 18.0, 33.3, 55.8, 56.5, 64.4, 114.9, 116.1, 125.7, 133.8, 139.8, 152.8.

(2R,4S)-4-Methyl-2-(phenylamino)-5-hexenol (**1j**). (a mixture of major and minor isomers in a ratio of 8:1): IR (neat) 3393 (s), 3078 (m), 2926 (s), 1601 (s), 1506 (s), 1317 (s), 1030 (s), 914 (m), 748 (s), 692 (s) cm^{-1}; ^{1}H-NMR ($CDCl_3$, major-isomer) δ 1.03 (d, J = 6.8 Hz, 3 H), 1.52 (t, J = 5.7 Hz, 2 H), 2.32 (ddm, J = 7.7, 6.8 Hz, 1 H), 3.49 (dd, J = 10.5, 5.4 Hz, 1 H), 3.55 (tdm, J = 5.4, 4.1 Hz, 2 H), 3.71 (dd, J = 10.5, 4.1 Hz, 1 H), 4.89 (dd, J = 17.2, 0.9 Hz, 1 H), 4.92 (dd, J = 10.4, 0.9 Hz, 1 H), 5.64 (ddd, J = 17.2, 10.4, 7.7 Hz, 1 H), 6.64 (dd, J = 8.6, 1.2 Hz, 2 H), 6.70 (t, J = 7.3 Hz, 1 H), 7.15 (dd, J = 8.6, 7.3 Hz, 2 H); ^{13}C-NMR ($CDCl_3$, major-isomer) δ 21.1, 35.0, 39.8, 53.5, 65.0, 113.7, 113.8,

117.8, 129.3, 143.7, 147.7; ^{1}H-NMR ($CDCl_3$, minor-isomer) δ 1.00 (d, *J* = 6.8 Hz, 3 H), 1.50 (t, *J* = 5.1 Hz, 2 H), 2.32 (ddm, *J* = 7.7, 6.8 Hz, 1 H), 3.49 (dd, *J* = 10.5, 5.4 Hz, 1 H), 3.55 (tdm, *J* = 5.4, 4.1 Hz, 2 H), 3.71 (dd, *J* = 10.5, 4.1 Hz, 1 H), 4.89 (dd, *J* = 17.2, 0.9 Hz, 1 H), 4.92 (dd, *J* = 10.4, 0.9 Hz, 1 H), 5.64 (ddd, *J* = 17.2, 10.4, 7.7 Hz, 1 H), 6.64 (dd, *J* = 8.6, 1.2 Hz, 2 H), 6.70 (t, *J* = 7.3 Hz, 1 H), 7.14 (dd, *J* = 8.5, 7.3 Hz, 2 H); ^{13}C-NMR ($CDCl_3$, minor-isomer) δ 21.1, 35.0, 39.8, 53.5, 65.0, 113.7, 113.8, 117.8, 129.3, 143.7, 147.7; HRMS, calcd for $C_{13}H_{19}NO$: 205.1467. Found *m/z* (relative intensity): 206.1505 (M^++1, 4), 205.1463 (M^+, 20), 175.1278 (16), 174.1268 (100).

(2R,4S)-2-(4-Methoxyphenylamino)-4-methyl-5-hexenol (**1k**). IR (neat) 3383 (s), 3078 (m), 2932 (s), 1618 (s), 1418 (s), 1238 (s), 1040 (s), 914 (m), 820 (s), 667 (m) cm^{-1}; ^{1}H-NMR ($CDCl_3$) δ 1.01 (d, *J* = 6.6 Hz, 3 H), 1.49 (t, *J* = 6.8 Hz, 2 H), 2.31 (qtm, *J* = 6.8, 6.6 Hz, 1 H), 3.45 (dd, *J* = 8.4, 3.2 Hz, 1 H), 3.46 (dd, *J* = 8.4, 5.5 Hz, 1 H), 3.71 (dm, *J* = 6.6 Hz, 1 H), 3.74 (s, 3 H), 4.89 (dd, *J* = 17.1, 1.2 Hz, 1 H), 4.92 (dd, *J* = 10.2, 1.2 Hz, 1 H), 5.63 (ddd, *J* = 17.1, 10.2, 8.1 Hz, 1 H), 6.64 (dd, *J* = 6.6, 2.2 Hz, 2 H), 6.76 (dd, *J* = 6.6, 2.2 Hz, 2 H); ^{13}C-NMR ($CDCl_3$) δ 21.0, 34.9, 39.6, 55.0, 55.7, 64.8, 113.6, 114.8, 115.4, 141.7, 143.7, 152.3; HRMS, calcd for $C_{14}H_{21}NO_2$: 235.1572. Found *m/z* (relative intensity): 236.1583 (M^++1, 5), 235.1562 (M^+, 28), 204.1378 (100).

(2R,4S)-2-(2-Methoxyphenylamino)-4-methyl-5-hexenol (**1l**). (a mixture of major and minor isomers in a ratio of 8:1): IR (neat) 3414 (s), 3070 (m), 2932 (s), 2359 (s), 1601 (s), 1516 (s), 1456 (s), 1223 (s), 1030 (s), 914 (s), 737 (s) cm^{-1}; ^{1}H-NMR ($CDCl_3$, major-isomer) δ 1.03 (d, *J* = 6.8 Hz, 3 H), 1.54 (t, *J* = 6.6 Hz, 2 H), 2.31 (qm, *J* = 6.8 Hz, 1 H), 3.50 (dd, *J* = 10.4, 5.8 Hz, 1 H), 3.55 (tdm, *J* = 6.6, 3.9 Hz, 1 H), 3.72 (dd, *J* = 10.4, 3.9 Hz, 1 H), 3.85 (s, 3 H), 4.87 (dd, *J* = 17.8, 1.7 Hz, 1 H), 4.90 (dd, *J* = 10.1, 1.7 Hz, 1 H), 5.64 (ddd, *J* = 17.8, 10.1, 8.0 Hz, 1 H), 6.68 (d, *J* = 7.6 Hz, 1 H), 6.69 (dd, *J* = 7.6, 1.6 Hz, 1 H), 6.77 (dd, *J* = 7.6, 1.2 Hz, 1 H), 6.83 (dm, *J* = 7.6 Hz, 1 H); ^{13}C-NMR ($CDCl_3$, major-isomer) δ 21.1, 34.9, 39.8, 53.4, 55.5, 65.1, 109.8, 111.2, 113.7, 116.9, 121.3, 137.4, 143.7, 147.0; ^{1}H-NMR ($CDCl_3$, minor-isomer) δ 0.99 (d, *J* = 6.8 Hz, 3 H), 1.54 (t, *J* = 6.6 Hz, 2 H), 2.31 (qm, *J* = 6.8 Hz, 1 H), 3.50 (dd, *J* = 10.4, 5.8 Hz, 1 H), 3.55 (tdm, *J* = 6.6, 3.9 Hz, 1 H), 3.72 (dd, *J* = 10.4, 3.9 Hz, 1 H), 3.85 (s, 3 H), 4.87 (dd, *J* = 17.8, 1.7 Hz, 1 H), 4.90 (dd, *J* = 10.1, 1.7 Hz, 1 H), 5.64 (ddd, *J* = 17.8, 10.1, 8.0 Hz, 1 H), 6.68 (d, *J* = 7.6 Hz, 1 H), 6.69 (dd, *J* = 7.6, 1.6 Hz, 1 H), 6.77 (dd, *J* = 7.6, 1.2 Hz, 1 H), 6.83 (dm, *J* = 7.6 Hz, 1 H); ^{13}C-NMR ($CDCl_3$, minor-isomer) δ 21.1, 35.0, 39.8, 53.4, 55.6, 65.1, 109.8, 111.2, 113.7, 116.9, 121.3, 137.4, 143.8, 147.0; HRMS, calcd for $C_{14}H_{21}NO_2$: 235.1572. Found *m/z* (relative intensity): 235.1568 (M^+, 29), 205.1415 (19), 204.1378 (100).

(2R,4S)-2-(4-Bromophenylamino)-4-methyl-5-hexenol (**1m**). IR (neat) 3400 (s), 2927 (s), 2868 (s), 2362 (m), 2345 (s), 1593 (s), 1496 (s), 1317 (s), 1074 (s), 916 (m), 812 (s) cm^{-1}; ^{1}H-NMR ($CDCl_3$) δ1.01 (d, *J* = 6.6 Hz, 3 H), 1.50 (m, 2 H), 2.02 (br, 1 H), 2.30 (m, 1 H), 3.48 (m, 2 H), 3.71 (dd, *J* = 13 6.2 Hz, 1 H), 4.88 (dd, *J* = 23, 1.2 Hz, 1 H), 4.92 (dd, *J* = 16, 1.2 Hz, 1 H), 5.62 (ddd, *J* = 17.0, 10.2, 8.1 Hz, 1 H), 6.49 (d, *J* = 9.0 Hz, 2 H), 7.21 (d, *J* = 9.0 Hz, 2 H); ^{13}C-NMR ($CDCl_3$) δ 21.0, 34.9, 38.5, 53.4, 64.8, 109.0, 113.9, 115.0, 131.8, 143.5, 146.7; HRMS, calcd for $C_{13}H_{18}BrNO$: 283.0572. Found *m/z* (relative intensity): 283.0562 (M^+, 25), 254.0476 (100).

(2R,4S)-2-(4-Methoxyphenylamino)-8-methyl-4-vinylnon-7-enol (**1n**). IR (neat) 3368 (s), 3078 (m), 2916 (s), 1607 (m), 1514 (s), 1375 (s), 1240 (s), 1042 (s), 914 (s), 820 (s) cm^{-1}; ^{1}H-NMR ($CDCl_3$) δ

1.33 (m, 2 H), 1.46 (dd, *J* = 10.1, 4.4 Hz, 2 H), 1.56 (s, 3 H), 1.67 (s, 3 H), 1.94 (m, 2 H), 2.15 (dm, *J* = 4.4 Hz, 1 H), 3.44 (dd, *J* = 10.0, 7.4 Hz, 1 H), 3.46 (dd, *J* = 10.0, 5.8 Hz, 1 H), 3.69 (m, 2 H), 3.74 (s, 3 H), 4.84 (dd, *J* = 17.0, 2.0 Hz, 1 H), 4.99 (dd, *J* = 10.2, 2.0 Hz, 1 H), 5.05 (tt, *J* = 5.6, 1.4 Hz, 1 H), 5.49 (ddd, *J* = 17.0, 10.2, 9.0 Hz, 1 H), 6.65 (dd, *J* = 6.6, 2.3 Hz, 2 H), 6.75 (dd, *J* = 6.6, 2.3 Hz, 2 H); ^{13}C-NMR ($CDCl_3$) δ 17.7, 25.6, 25.7, 35.5, 38.1, 40.5, 55.7, 65.0, 114.8, 115.5, 115.8, 124.1, 131.4, 141.2, 142.2, 152.7; HRMS, calcd for $C_{19}H_{29}NO_2$: 303.2198. Found *m/z* (relative intensity): 304.2201 (M^+ +1, 11), 303.2183 (M^+, 48), 273.2022 (19), 272.2000 (100).

3-(4-Methoxyphenylamino)-6-hexene-1,2-diol (**1o**). (a mixture of major and minor isomers in a ratio of 3:1): IR (neat) 3356 (s), 3074 (m), 2934 (s), 1666 (s), 1441 (s), 1236 (s), 1038 (s), 822 (s) cm^{-1}; ^{1}H-NMR ($CDCl_3$, major-isomer) δ 1.62 (td, *J* = 8.7, 5.9 Hz, 2 H), 2.00–2.33 (m, 2 H), 2.60–3.00 (m, 1 H), 3.35–3.47 (m, 1 H), 3.68–3.80 (m, 2 H), 3.74 (s, 3 H), 4.95 (dd, *J* = 9.9, 1.5 Hz, 1 H), 4.96 (dd, *J* = 17.8, 1.5 Hz, 1 H), 5.76 (ddt, *J* = 17.8, 9.9, 6.7 Hz, 1 H), 6.65 (dt, *J* = 9.3, 2.5 Hz, 2 H), 6.76 (dt, *J* = 9.3, 2.5 Hz, 2 H); ^{13}C-NMR ($CDCl_3$, major-isomer) δ 30.3, 30.8, 55.7, 57.8, 64.0, 72.7, 114.9, 115.2, 115.6, 137.7, 141.1, 152.6; ^{1}H-NMR ($CDCl_3$, minor-isomer) δ 1.62 (td, *J* = 8.7, 5.9 Hz, 2 H), 2.00–2.33 (m, 2 H), 2.60–3.00 (m, 1 H), 3.35–3.47 (m, 1 H), 3.68–3.80 (m, 2 H), 3.74 (s, 3 H), 4.95 (dd, *J* = 9.9, 1.5 Hz, 1 H), 4.96 (dd, *J* = 17.8, 1.5 Hz, 1 H), 5.76 (ddt, *J* = 17.8, 9.9, 6.7 Hz, 1 H), 6.65 (dt, *J* = 9.3, 2.5 Hz, 2 H), 6.76 (dt, *J* = 9.3, 2.5 Hz, 2 H); ^{13}C-NMR ($CDCl_3$, minor-isomer) δ 30.2, 30.8, 55.7, 57.8, 64.2, 72.6, 114.8, 115.2, 115.6, 137.7, 141.2, 152.6; HRMS, calcd for $C_{14}H_{21}NO_3$: 251.1521. Found *m/z* (relative intensity): 252.1614 (M^++1, 4), 251.1512 (M^+, 30), 220.1334 (6), 191.1258 (14), 190.1187 (100).

(E)-3-(4-Methoxyphenylamino)-5-hexene-1,2-diol (**1o'**). (a mixture of major and minor isomers in a ratio of 3:1): ^{1}H-NMR ($CDCl_3$, major-isomer) δ 1.72 (d, *J* = 6.8 Hz, 3 H), 2.00–2.33 (m, 2 H), 2.60–3.00 (m, 1 H), 3.35–3.47 (m, 1 H), 3.68–3.80 (m, 2 H), 3.74 (s, 3 H), 5.36–5.43 (m, 1 H), 5.48–5.55, (m, 1 H), 6.65 (dt, *J* = 9.3, 2.5 Hz, 2 H), 6.76 (dt, *J* = 9.3, 2.5 Hz, 2 H); ^{13}C-NMR ($CDCl_3$, major-isomer) δ 18.0, 33.8, 55.7, 57.4, 64.8, 72.2, 114.9, 115.2, 126.5, 128.8, 141.5, 152.8; ^{1}H-NMR ($CDCl_3$, minor-isomer) δ 1.72 (d, *J* = 6.8 Hz, 3 H), 2.00–2.33 (m, 2 H), 2.60–3.00 (m, 1 H), 3.35–3.47 (m, 1 H), 3.68–3.80 (m, 2 H), 3.74 (s, 3 H), 5.36–5.43 (m, 1 H), 5.48–5.55, (m, 1 H), 6.65 (dt, *J* = 9.3, 2.5 Hz, 2 H), 6.76 (dt, *J* = 9.3, 2.5 Hz, 2 H); ^{13}C-NMR ($CDCl_3$, minor-isomer) δ 18.0, 33.8, 55.7, 57.4, 64.7, 72.2, 114.8, 115.2, 126.5, 128.8, 141.5, 152.8.

(3R,5S)-5-Methyl-3-(phenylamino)-6-heptene-1,2-diol (**1p**). (a mixture of major and minor isomers in a ratio of 2:1): IR (neat) 3281 (s), 2961 (s), 1603 (s), 1512 (s), 1325 (s), 1024 (s), 748 (s), 692 (s) cm^{-1}; ^{1}H-NMR ($CDCl_3$, major-isomer) δ 1.01 (d, *J* = 6.8 Hz, 3 H), 1.54–1.63 (m, 2 H), 2.23–2.33 (qm, *J* = 6.8 Hz, 1 H), 3.54–3.77 (m, 4 H), 4.94 (dd, *J* = 10.3, 1.5 Hz, 1 H), 4.98 (dd, *J* = 17.1, 1.5 Hz, 1 H), 5.59 (ddd, *J* = 17.1, 10.3, 8.3 Hz, 1 H), 6.65 (td, *J* = 8.5, 1.0 Hz, 2 H), 6.70–6.74 (m, 1 H), 7.15 (dt, *J* = 8.5, 7.6 Hz, 2 H); ^{13}C-NMR ($CDCl_3$, major-isomer) δ 21.4, 34.9, 39.0, 54.3, 64.0, 73.5, 113.7, 117.8, 118.1, 129.2, 143.4, 147.6; ^{1}H-NMR ($CDCl_3$, minor-isomer) δ 0.97 (d, *J* = 6.6 Hz, 3 H), 1.47 (ddd, *J* = 14.0, 9.8, 4.2 Hz, 2 H), 2.23–2.33 (qm, *J* = 6.8 Hz, 1 H), 3.54–3.77 (m, 4 H), 4.82 (dd, *J* = 17.3, 1.7 Hz, 1 H), 4.92 (dd, *J* = 9.9, 1.7 Hz, 1 H), 5.72 (ddd, *J* = 17.3, 9.9, 7.4 Hz, 1 H), 6.65 (td, *J* = 8.5, 1.0 Hz, 2 H), 6.70–6.74 (m, 1 H), 7.15 (dt, *J* = 8.5, 7.6 Hz, 2 H); ^{13}C-NMR ($CDCl_3$,

minor-isomer) δ 21.2, 34.5, 38.2, 54.6, 63.8, 72.7, 113.8, 117.8, 118.0, 129.3, 143.9, 147.4; HRMS, calcd for $C_{14}H_{21}NO_2$: 235.1572. Found *m/z* (relative intensity): 236.1596 (M^++1, 3), 235.1554 (M^+, 12), 175.1262 (13), 174.1240 (100).

(3R,5S)-3-(4-Methoxyphenylamino)-5-methyl-6-heptene-1,2-diol (**1q**). (a mixture of major and minor isomers in a ratio of 2:1): IR (neat) 3366 (s), 3078 (m), 2932 (m), 2835 (m), 1655 (s), 1238 (s), 1036 (s), 822 (s) cm^{-1}; ^{1}H-NMR ($CDCl_3$, major-isomer) δ 1.00 (d, *J* = 6.6 Hz, 3 H), 1.48 (ddd, *J* = 14.0, 9.7, 4.2 Hz, 1 H), 1.55 (ddd, *J* = 14.0, 9.7, 4.2 Hz, 1 H), 2.03 (br, 2 H) 2.25 (m, 1 H), 3.44–3.54 (m, 1 H), 3.74 (s, 3 H), 3.66–3.8 (m, 4 H), 4.80 (dd, *J* = 17.1, 1.3 Hz, 1 H), 4.91 (dd, *J* = 10.5, 1.3 Hz, 1 H), 5.57 (ddd, *J* = 17.1, 10.5, 8.3 Hz, 1 H), 6.68 (dd, *j* = 6.8, 2.3 Hz, 2 H), 6.76 (dd, *J* = 6.8, 2.3 Hz, 2 H); ^{13}C-NMR ($CDCl_3$, major-isomer) δ 21.4, 35.0, 39.0, 55.8, 56.3, 64.1, 73.1, 114.2, 114.9, 115.9, 143.5, 143.7, 152.8; ^{1}H-NMR ($CDCl_3$, minor-isomer) δ 0.96 (d, *J* = 6.6 Hz, 3 H), 1.48 (ddd, *J* = 14.0, 9.7, 4.2 Hz, 1 H), 1.55 (ddd, *J* = 14.0, 9.7, 4.2 Hz, 1 H), 2.03 (br, 2 H) 2.25 (m, 1 H), 3.44–3.54 (m, 1 H), 3.74 (s, 3 H), 3.66–3.8 (m, 4 H), 4.80 (dd, *J* = 17.1, 1.3 Hz, 1 H), 4.97 (dd, *J* = 17.4, 1.3 Hz, 1 H), 5.67 (ddd, *J* = 17.4, 10.2, 7.6 Hz, 1 H), 6.68 (dd, *J* = 6.8, 2.3 Hz, 2 H), 6.76 (dd, *J* = 6.8, 2.3 Hz, 2 H); ^{13}C-NMR ($CDCl_3$, minor-isomer) δ 20.4, 34.7, 39.0, 55.8, 56.3, 63.8, 73.1, 115.0, 114.9, 116.1, 143.5, 143.7, 152.8; HRMS, calcd for $C_{15}H_{23}NO_3$: 265.1678. Found *m/z* (relative intensity): 266.1678 (M^++1, 3), 265.1659 (M^+, 18), 205.1406 (15), 204.1406 (100).

(3R,5S)-3-(4-Methoxyphenylamino)-9-methyl-5-vinyl-8-decene-1,2-diol (**1r**). (a mixture of major and minor isomers in a ratio of 2:1): IR (neat) 3358 (s), 3074 (s), 2916 (s), 2343 (m), 1666 (s), 1514 (s), 1238 (s), 1040 (s), 822 (s) cm^{-1}; ^{1}H-NMR ($CDCl_3$, major-isomer) δ 1.19–1.38 (m, 4 H), 1.55 (s, 3 H), 1.67 (s, 3 H), 1.79–1.98 (dm, *J* = 7.5 Hz, 1 H), 2.00–2.17 (m, 2 H), 3.47 (dd, *J* = 12.0, 5.9 Hz, 1 H), 3.54 (dd, *J* = 12.0, 6.9 Hz, 1 H), 3.69–3.80 (m, 3 H), 3.74 (s, 3 H), 4.74 (dd, *J* = 18.6, 2.0 Hz, 1 H), 4.94–5.05 (m, 1 H), 4.98 (dd, *J* = 10.0, 2.0 Hz, 1 H), 5.46 (ddd, *J* = 18.6, 10.0, 7.5 Hz, 1 H), 6.69 (d, *J* = 6.6 Hz, 2 H), 6.75–6.78 (m, 2 H); ^{13}C-NMR ($CDCl_3$, major-isomer) δ 17.7, 25.4, 25.6, 35.2, 35.4, 40.5, 55.6, 55.7, 64.0, 73.1, 114.9, 115.3, 116.2, 124.1, 124.3, 131.5, 131.6, 141.9; ^{1}H-NMR ($CDCl_3$, minor-isomer) δ 1.19–1.38 (m, 4 H), 1.54 (s, 3 H), 1.64 (s, 3 H), 1.79–1.98 (dm, *J* = 7.5 Hz, 1 H), 2.00–2.17 (m, 2 H), 3.47 (dd, *J* = 12.0, 5.9 Hz, 1 H), 3.54 (dd, *J* = 12.0, 6.9 Hz, 1 H), 3.69–3.80 (m, 3 H), 3.75 (s, 3 H), 4.74 (dd, *J* = 18.6, 2.0 Hz, 1 H), 4.94–5.05 (m, 1 H), 4.98 (dd, *J* = 10.0, 2.0 Hz, 1 H), 5.46 (ddd, *J* = 18.6, 10.0, 7.5 Hz, 1 H), 6.69 (d, *J* = 6.6 Hz, 2 H), 6.75–6.78 (m, 2 H); ^{13}C-NMR ($CDCl_3$, minor-isomer) δ 17.7, 25.4, 25.6, 35.2, 35.4, 40.4, 55.6, 55.7, 64.0, 73.1, 114.8, 115.3, 116.3, 124.1, 124.3, 131.4, 131.6, 141.8; HRMS, calcd for $C_{20}H_{31}NO_3$: 333.2304. Found *m/z* (relative intensity): 334.2348 (M^++1, 7), 333.2312 (M^+, 33), 302.2102 (4), 273.2012 (20), 272.2004 (100).

(2S, 3S, 5S)-5-(4-Methoxyphenylamino)-8-nonen-1,2,3-triol (**1s**). (a mixture of major and minor isomers in a ratio of 1:1): IR (neat) 3277 (m), 2932 (s), 2839 (s), 1732 (s), 1514 (s), 1456 (s), 1441 (s), 1238 (s), 1040 (s), 970 (s), 912 (m), 824 (s) cm^{-1}; ^{1}H-NMR ($CDCl_3$, major-isomer) δ 1.43 (m, 2 H), 1.87 (dt, *J* = 11.5, 3.2 Hz, 2 H), 2.15–2.30 (br d, *J* = 6.8 Hz, 2 H), 3.51–3.62 (m, 4 H), 3.61 (br t, *J* = 3.2 Hz, 1 H), 3.74 (s, 3 H), 3.93–4.01 (m, 1 H), 4.95 (dd, *J* = 11.2, 1.6 Hz, 1 H), 4.98 (dd, *J* = 18.1, 1.6 Hz, 1 H), 5.78 (ddd, *J* = 18.1, 11.2, 6.8 Hz, 1 H), 6.70 (d, *J* = 8.8 Hz, 2 H), 6.77 (d, *J* = 8.8 Hz, 2 H); ^{13}C-NMR ($CDCl_3$, major-isomer) δ 28.7, 32.7, 36.5, 55.7, 56.8, 63.7, 71.3, 74.6, 114.5, 114.8,

118.3, 138.5, 141.1, 153.2; ^{1}H-NMR ($CDCl_3$, minor-isomer) δ 1.43 (m, 2 H), 1.87 (dt, *J* = 11.5, 3.2 Hz, 2 H), 2.15–2.30 (br d, *J* = 6.8 Hz, 2 H), 3.51–3.62 (m, 4 H), 3.61 (br t, *J* = 3.2 Hz, 1 H), 3.74 (s, 3 H), 3.93–4.01 (m, 1 H), 4.95 (dd, *J* = 11.2, 1.6 Hz, 1 H), 4.98 (dd, *J* = 18.1, 1.6 Hz, 1 H), 5.78 (ddd, *J* = 18.1, 11.2, 6.8 Hz, 1 H), 6.70 (d, *J* = 8.8 Hz, 2 H), 6.77 (d, *J* = 8.8 Hz, 2 H); ^{13}C-NMR ($CDCl_3$, minor-isomer) δ 28.7, 32.7, 36.1, 55.7, 56.8, 63.7, 71.3, 74.7, 114.6, 114.8, 118.5, 138.5, 141.1, 153.3; HRMS, calcd for $C_{16}H_{25}NO_4$: 295.1783. Found *m*/*z* (relative intensity): 296.1817 (M^++1, 21), 295.1776 (M^+, 100), 294.1700 (5).

(2S, 3S, 5S)-5-(4-Methoxyphenylamino)-7-nonen-1,2,3-triol (**1s'**). (a mixture of major and minor isomers in a ratio of 3:1): ^{1}H-NMR ($CDCl_3$, major-isomer) δ 1.43 (m, 2 H), 1.87 (dt, *J* = 11.5, 3.2 Hz, 2 H), 2.15–2.30 (m, 2 H), 2.03 (d, *J* = 7.3 Hz, 3 H), 3.51–3.62 (m, 4 H), 3.61 (br t, *J* = 3.2 Hz, 1 H), 3.74 (s, 3 H), 3.93–4.01 (m, 1 H), 5.32 (dq, *J* = 14.6, 7.3 Hz, 1 H), 5.46 (dt, *J* = 14.6, 6.6 Hz, 1 H), 6.70 (d, *J* = 8.8 Hz, 2 H), 6.77 (d, *J* = 8.8 Hz, 2 H); ^{13}C-NMR ($CDCl_3$, major-isomer) δ 18.0, 32.5, 37.7, 52.7, 55.7, 63.9, 71.1, 74.1, 114.9, 116.9, 125.5, 130.6, 140.8, 154.0; ^{1}H-NMR ($CDCl_3$, minor-isomer) δ 1.43 (m, 2 H), 1.87 (dt, *J* = 11.5, 3.2 Hz, 2 H), 2.15–2.30 (m, 2 H), 2.03 (d, *J* = 7.3 Hz, 3 H), 3.51–3.62 (m, 4 H), 3.61 (br t, *J* = 3.2 Hz, 1 H), 3.74 (s, 3 H), 3.93–4.01 (m, 1 H), 5.32 (dq, *J* = 14.6, 7.3 Hz, 1 H), 5.46 (dt, *J* = 14.6, 6.6 Hz, 1 H), 6.70 (d, *J* = 8.8 Hz, 2 H), 6.77 (d, *J* = 8.8 Hz, 2 H); ^{13}C-NMR ($CDCl_3$, minor-isomer) δ 18.0, 32.5, 37.4, 52.7, 55.7, 63.9, 71.1, 74.3, 114.9, 117.0, 125.2, 130.7, 140.8, 154.0.

(2S, 3S, 5S, 7S)-5-(4-Methoxyphenylamino)-7-methyl-8-nonen-1,2,3-triol (**1t**). (a mixture of major and minor isomers in a ratio of 2:1): IR (neat) 3267 (s), 2835 (s), 1639 (m), 1616 (m), 1417 (s), 1238 (s), 1180 (s), 1038 (s), 914 (s), 824 (s), 606 (s) cm^{-1}; ^{1}H-NMR ($CDCl_3$, major-isomer) δ 1.00 (d, *J* = 6.6 Hz, 3 H), 1.46–1.64 (m, 4 H), 1.88 (ddd, *J* = 14.4, 8.5, 3.4 Hz, 1 H), 2.26–2.33 (br-d, *J* = 7.3 Hz, 1 H), 3.48–3.80 (m, 3 H), 3.75 (s, 3 H), 3.97 (ddd, *J* = 11.5, 5.6, 2.9 Hz, 1 H), 4.89 (dd, *J* = 17.2, 1.0 Hz, 1 H), 4.91 (dd, *J* = 9.7, 1.7 Hz, 1 H), 5.65 (ddd, *J* = 17.2, 9.7, 7.3 Hz, 1 H), 6.66 (d, *J* = 9.0 Hz, 2 H), 6.77 (d, *J* = 9.0 Hz, 2 H); ^{13}C-NMR ($CDCl_3$, major-isomer) δ 20.7, 35.1, 37.4, 42.6, 51.2, 55.8, 63.9, 71.3, 74.0, 113.5, 114.9, 116.2, 141.0, 144.0, 153.0; ^{1}H-NMR ($CDCl_3$, minor-isomer) δ 0.94 (d, *J* = 6.8 Hz, 3 H), 1.46–1.64 (m, 4 H), 1.91 (ddd, *J* = 14.4, 8.5, 3.4 Hz, 1 H), 2.26–2.33 (br-d, *J* = 7.3 Hz, 1 H), 3.48–3.80 (m, 3 H), 3.75 (s, 3 H), 3.97 (ddd, *J* = 11.5, 5.6, 2.9 Hz, 1 H), 4.89 (dd, *J* = 17.2, 1.0 Hz, 1 H), 4.91 (dd, *J* = 9.7, 1.7 Hz, 1 H), 5.65 (ddd, *J* = 17.2, 9.7, 7.3 Hz, 1 H), 6.67 (d, *J* = 10.5 Hz, 2 H), 6.69 (d, *J* = 10.2 Hz, 2 H); ^{13}C-NMR ($CDCl_3$, minor-isomer) δ 20.7, 34.9, 37.2, 43.2, 51.6, 55.7, 63.8, 71.3, 74.2, 113.5, 114.9, 116.2, 141.0, 144.2, 153.0; HRMS, calcd for $C_{17}H_{27}NO_4$: 309.1940. Found *m*/*z* (relative intensity): 310.1951 (M^++1, 19), 309.1932 (M^+, 100), 248.1652 (12), 247.1566 (5).

(2S, 3S, 5S, 7S)-5-(2-Methoxyphenylamino)-7-methyl-8-nonen-1,2,3-triol (**1u**). (a mixture of major and minor isomers in a ratio of 2:1): IR (neat) 3379 (s), 3071 (s), 2932 (s), 2360 (s), 1596 (s), 1512 (s), 1458 (s), 1227 (s), 1026 (s), 910 (s), 733 (s), 679 (s) cm^{-1}; ^{1}H-NMR ($CDCl_3$, major-isomer) δ 0.99 (d, *J* = 6.6 Hz, 3 H), 1.42–1.52 (br d, *J* = 6.6 Hz, 4 H), 2.22–2.27 (br d, *J* = 6.6 Hz, 1 H), 3.42–3.43 (m, 1 H), 3.57–3.58 (m, 2 H), 3.76–3.78 (m, 3 H), 3.77 (s, 3 H), 4.01 (br, 3 H), 4.83 (d, *J* = 17.3 Hz, 1 H), 4.86 (d, *J* = 10.0 Hz, 1 H), 5.62 (ddd, *J* = 17.3, 10.0, 7.5 Hz, 1 H), 6.56–6.64 (m, 1 H), 6.70–6.72 (m,

2 H), 6.78 (t, *J* = 7.6 Hz, 1 H); ^{13}C-NMR ($CDCl_3$, major-isomer) δ 20.5, 34.7, 38.4, 43.3, 48.4, 55.3, 63.1, 70.1, 74.5, 109.7, 110.8, 113.1, 116.2, 121.2, 137.6, 143.9, 146.6; ^{1}H-NMR ($CDCl_3$, minor-isomer) δ 0.95 (d, *J* = 6.3 Hz, 3 H), 1.65–1.78 (m, 4 H), 2.22–2.27 (br d, *J* = 6.6 Hz, 1 H), 3.42–3.43 (m, 1 H), 3.57–3.58 (m, 2 H), 3.76–3.78 (m, 3 H), 3.77 (s, 3 H), 4.01 (br, 3 H), 4.83 (d, *J* = 17.3 Hz, 1 H), 4.86 (d, *J* = 10.0 Hz, 1 H), 5.62 (ddd, *J* = 17.3, 10.0, 7.5 Hz, 1 H), 6.56–6.64 (m, 1 H), 6.70–6.72 (m, 2 H), 6.78 (t, *J* = 7.6 Hz, 1 H); ^{13}C-NMR ($CDCl_3$, minor-isomer) δ 20.7, 34.6, 38.4, 43.3, 48.2, 55.3, 63.1, 70.1, 74.5, 109.7, 110.8, 113.2, 116.4, 121.2, 137.5, 143.9, 146.6; HRMS, calcd for $C_{17}H_{27}NO_4$: 309.1940. Found *m*/*z* (relative intensity): 309.1922 (M^+, 100).

(2S, 3S, 5S, 7S)-5-(3,4-Dimethoxyphenylamino)-7-methyl-8-nonen-1,2,3-triol (**1v**). (a mixture of major and minor isomers in a ratio of 2:1):IR (neat) 3379 (s), 3078 (m), 2932 (s), 1705 (m), 1612 (s), 1458 (s), 1234 (s), 1026 (s), 918 (m), 733 (s) cm^{-1}; ^{1}H-NMR ($CDCl_3$, major-isomer) δ 1.00 (d, *J* = 6.6 Hz, 3 H), 1.45–1.63 (m, 4 H), 1.86 (ddd, *J* = 14.2, 9.3, 3.2 Hz, 1 H), 2.20–2.40 (m, 1 H), 3.12 (br, 1 H), 3.56 (br dd, *J* = 9.5, 5.4 Hz, 1 H), 3.75 (m, 2 H), 3.80 (s, 3 H), 3.82 (s, 3 H), 3.95 (ddd, *J* = 8.8, 5.4, 3.1 Hz, 1 H), 4.90 (d, *J* = 17.1 Hz, 1 H), 4.92 (d, *J* = 10.5 Hz, 1 H), 5.65 (ddd, *J* = 17.1, 10.5, 7.9 Hz, 1 H), 6.23 (dd, *J* = 8.5, 2.4 Hz, 1 H), 6.32 (d, *J* = 2.4 Hz, 1 H), 6.72 (d, *J* = 8.5 Hz, 1 H); ^{13}C-NMR ($CDCl_3$, major-isomer) δ 20.7, 35.0, 37.7, 42.7, 50.8, 55.8, 56.6, 56.8, 63.6, 70.9, 100.5, 105.7, 113.4, 122.3, 132.3, 141.7, 144.0, 149.9; ^{1}H-NMR ($CDCl_3$, minor-isomer) δ 0.96 (d, *J* = 6.8 Hz, 3 H), 1.45–1.63 (m, 4 H), 1.95 (ddd, *J* = 14.4, 8.8, 3.2 Hz, 1 H), 2.20–2.40 (m, 1 H), 3.12 (br, 1 H), 3.56 (br dd, *J* = 9.5, 5.4 Hz, 1 H), 3.75 (m, 2 H), 3.80 (s, 3 H), 3.82 (s, 3 H), 4.00 (m, 1 H), 4.88 (d, *J* = 18.8 Hz, 1 H), 4.98 (br d, *J* = 10.8 Hz, 1 H), 5.58 (ddd, *J* = 18.8, 10.8, 8.0 Hz, 1 H), 6.29 (dt, *J* = 8.5, 2.4 Hz, 1 H), 6.34 (d, *J* = 2.4 Hz, 1 H), 6.74 (d, *J* = 8.5 Hz, 1 H); ^{13}C-NMR ($CDCl_3$, minor-isomer) δ 20.7, 35.0, 37.7, 42.7, 50.8, 55.8, 56.6, 56.8, 63.6, 70.9, 100.5, 105.7, 113.4, 122.3, 132.3, 141.7, 144.0, 149.9; HRMS, calcd for $C_{18}H_{29}NO_5$: 339.2046. Found *m*/*z* (relative intensity): 340 (M^++1, 24), 339.2039 (M^+, 100), 324 (7), 321 (3).

(2S, 3S, 5S, 7S)-5-Phenylamino-7-methyl-8-nonen-1,2,3-triol (**1w**). (a mixture of major and minor isomers in a ratio of 2:1): IR (neat) 3400 (s), 2870 (s), 1639 (s), 1602 (s), 1502 (s), 1259 (s), 1180 (s), 993 (s), 873 (s), 754 (s), 667 (s) cm^{-1}; ^{1}H-NMR ($CDCl_3$, major-isomer) δ 1.01 (d, *J* = 6.8 Hz, 3 H), 1.52–1.58 (m, 4 H), 1.85 (ddd, *J* = 14.4, 9.3, 3.4 Hz, 2 H), 2.28–2.35 (br d, *J* = 7.2 Hz, 1 H), 3.55 (br d, *J* = 9.3 Hz, 1 H), 3.77 (m, 3 H), 3.95 (ddd, *J* = 9.3, 5.4, 2.7 Hz, 1 H), 4.88 (dt, *J* = 17.4, 1.0 Hz, 1 H), 4.92 (dt, *J* = 9.9, 0.9 Hz, 1 H), 5.65 (ddd, *J* = 17.4, 9.9, 7.5 Hz, 1 H), 6.65 (dd, *J* = 8.5, 1.0 Hz, 2 H), 6.70 (dd, *J* = 8.5, 7.3 Hz, 1 H), 7.16 (dd, *J* = 8.5, 7.3 Hz, 2 H); ^{13}C-NMR ($CDCl_3$, major-isomer) δ 20.9, 35.1, 38.1, 43.1, 49.5, 63.7, 71.0, 74.1, 113.9, 114.5, 129.3, 144.0, 147.5; ^{1}H-NMR ($CDCl_3$, minor-isomer) δ 0.97 (d, *J* = 6.8 Hz, 3 H), 1.52–1.58 (m, 4 H), 1.85 (ddd, *J* = 14.4, 9.3, 3.4 Hz, 2 H), 2.28–2.35 (br d, *J* = 7.2 Hz, 1 H), 3.55 (br d, *J* = 9.3 Hz, 1 H), 3.77 (m, 3 H), 3.95 (ddd, *J* = 9.3, 5.4, 2.7 Hz, 1 H), 4.88 (dt, *J* = 17.4, 1.0 Hz, 1 H), 4.92 (dt, *J* = 9.9, 0.9 Hz, 1 H), 5.59 (br dd, *J* = 9.6, 7.5 Hz, 1 H), 6.65 (dd, *J* = 8.5, 1.0 Hz, 2 H), 6.70 (dd, *J* = 7.6, 6.8 Hz, 1 H), 7.16 (dd, *J* = 8.5, 7.3 Hz, 2 H); ^{13}C-NMR ($CDCl_3$, minor-isomer) δ 20.9, 35.0, 38.1, 43.1, 49.5, 63.7, 71.0, 74.1, 113.6, 115.5, 129.3, 144.0, 147.5; HRMS, calcd for $C_{16}H_{25}NO_3$: 279.1834. Found *m*/*z* (relative intensity): 280.1877 (M^++1, 19), 279.1831 (M^+, 100), 278.1753 (4), 248.1628 (6), 217.1507 (3).

(2S, 3S, 5S, 7S)-4-(4-Chlorophenylamino)-7-methyl-8-nonen-1,2,3-triol (**1x**). (a mixture of major and minor isomers in a ratio of 2:1): IR (neat) 3364 (s), 3078 (s), 2924 (s), 2361 (s), 1705 (s), 1597 (s), 1504 (s), 1319 (s), 1258 (s), 1180 (s), 918 (s), 818 (s), 671 (s) cm^{-1}; ^{1}H-NMR ($CDCl_3$, major-isomer) δ 0.91 (d, *J* = 6.6 Hz, 3 H), 1.38 (t, *J* = 6.6 Hz, 2 H), 1.18–1.60 (m, 2 H), 2.18 (br quint, d, *J* = 6.6 Hz, 1 H), 3.34–3.39 (m, 1 H), 3.49–3.57 (m, 3 H), 3.70–3.72 (m, 2 H), 3.78 (br, 3 H), 4.76 (d, *J* = 17.2 Hz, 1 H), 4.83 (d, *J* = 10.2 Hz, 1 H), 5.52 (ddd, *J* = 17.2, 10.2, 7.9 Hz, 1 H), 6.46 (d, *J* = 8.8 Hz, 2 H), 6.97 (d, *J* = 8.8 Hz, 2 H); ^{13}C-NMR ($CDCl_3$, major-isomer) δ 20.0, 34.9, 38.4, 43.1, 49.2, 63.1, 70.2, 74.5, 113.6, 114.5, 121.6, 129.0, 143.9, 146.5; ^{1}H-NMR ($CDCl_3$, minor-isomer) δ 0.88 (d, *J* = 6.8 Hz, 3 H), 1.38 (t, *J* = 6.6 Hz, 2 H), 1.18–1.60 (m, 2 H), 2.18 (br quint, d, *J* = 6.6 Hz, 1 H), 3.34–3.39 (m, 1 H), 3.49–3.57 (m, 3 H), 3.70–3.72 (m, 2 H), 3.78 (br, 3 H), 4.76 (d, *J* = 17.2 Hz, 1 H), 4.83 (d, *J* = 10.2 Hz, 1 H), 5.52 (ddd, *J* = 17.2, 10.2, 7.9 Hz, 1 H), 6.51 (d, *J* = 8.8 Hz, 2 H), 7.02 (d, *J* = 8.8 Hz, 2 H); ^{13}C-NMR ($CDCl_3$, minor-isomer) δ 21.0, 34.8, 38.4, 42.7, 49.2, 63.1, 70.2, 74.4, 113.7, 114.5, 121.6, 129.1, 143.8, 146.5; HRMS, calcd for $C_{16}H_{24}ClNO_3$: 313.1445. Found *m*/*z* (relative intensity): 314.1446 (M^++1, 6), 313.1418 (M^+, 29), 245.0748 (13), 244.0726 (100).

(2S, 3S, 5S, 7S)-5-(4-Methoxyphenylamino)-7-vinyl-11-dodecen-1,2,3-triol (**1y**). (a mixture of major and minor isomers in a ratio of 1:1): IR (neat) 3300 (m), 2912 (s), 2835 (s), 1639 (s), 1618 (s), 1500 (s), 1456 (s), 1294 (s), 1238 (s), 1180 (s), 1039 (s), 916 (s), 821 (s), 748 (s) cm^{-1}; ^{1}H-NMR ($CDCl_3$, major-isomer) δ 1.18–1.36 (m, 2 H), 1.46–1.71 (m, 2 H), 1.56 (s, 3 H), 1.67 (s, 3 H), 1.82–2.00 (m, 2 H), 1.91 (ddd, *J* = 14.1, 9.2, 3.7 Hz, 2 H), 2.04–2.16 (m, 1 H), 3.53–3.59 (br dd, *J* = 9.2, 5.3 Hz, 1 H), 3.62–3.65 (br d, *J* = 3.7 Hz, 1 H), 3.71–3.85 (m, 2 H), 3.75 (s, 3 H), 3.95–4.00 (m, 1 H), 4.86 (dd, *J* = 17.1, 1.7 Hz, 1 H), 4.99 (dd, *J* = 10.1, 1.7 Hz, 1 H), 5.02–5.06 (t m, *J* = 7.1 Hz, 1 H), 5.49 (ddd, *J* = 17.1, 10.1, 7.1 Hz, 1 H), 6.67 (d, *J* = 8.9 Hz, 2 H), 6.76 (d, *J* = 8.9 Hz, 2 H); ^{13}C-NMR ($CDCl_3$, major-isomer) δ 17.8, 25.6, 25.8, 35.4, 37.6, 40.7, 41.1, 51.6, 55.8, 63.8, 71.4, 74.1, 114.9, 115.5, 116.5, 124.2, 131.5, 142.6, 142.8, 153.2; ^{1}H-NMR ($CDCl_3$, minor-isomer) δ 1.18–1.36 (m, 2 H), 1.46–1.71 (m, 2 H), 1.56 (s, 3 H), 1.67 (s, 3 H), 1.82–2.00 (m, 2 H), 1.91 (ddd, *J* = 14.1, 9.2, 3.7 Hz, 2 H), 2.04–2.16 (m, 1 H), 3.53–3.59 (br dd, *J* = 9.2, 5.3 Hz, 1 H), 3.62–3.65 (br d, *J* = 3.7 Hz, 1 H), 3.71–3.85 (m, 2 H), 3.75 (s, 3 H), 3.95–4.00 (m, 1 H), 4.80 (dd, *J* = 17.6, 1.6 Hz, 1 H), 4.97 (dd, *J* = 10.2, 1.6 Hz, 1 H), 5.02–5.06 (t m, *J* = 7.1 Hz, 1 H), 5.49 (ddd, *J* = 17.1, 10.1, 7.1 Hz, 1 H), 6.67 (d, *J* = 8.9 Hz, 2 H), 6.78 (d, *J* = 9.0 Hz, 2 H); ^{13}C-NMR ($CDCl_3$, minor-isomer) δ 17.8, 25.4, 25.7, 35.4, 37.6, 40.7, 41.1, 51.6, 55.7, 63.8, 71.4, 74.2, 114.8, 115.5, 116.5, 124.2, 131.5, 142.6, 142.8, 153.8; HRMS, calcd for $C_{22}H_{35}NO_4$: 377.2566. Found *m*/*z* (relative intensity): 377.2561 (M^+, 100).

General procedure for the Ni-catalyzed homoallylation of N,O-acetals prepared from carbohydrate and primary amines with dienes (entry 8, Table 4): A solution of D-ribose (150 mg, 1 mmol) and *p*-anisidine (246 mg, 2 mmol) in dry DMF (5 mL) was refluxed for 120 min under nitrogen. The solvent was removed by distillation under reduced pressure (azeotropic removal of water). A mixture of $Ni(cod)_2$ (27.5 mg, 0.1 mmol) and isoprene (800 μL, 8 mmol) dissolved in THF (2 mL) and triethylborane (6.0 mmol, 1.0 M THF solution) were successively added to the flask containing *N*,*O*-acetal. The reaction mixture was stirred at 50 °C for 48 h, and the reaction mixture was diluted with 30 mL of EtOAc and washed with sat. $NaHCO_3$, and brine. The organic phase was dried ($MgSO_4$) and concentrated in vacuo to give a colorless oil, which was subjected to column chromatography

over silica gel (hexane/EtOAc = 0/100 v/v) to give 2 (192 mg, 57%) in a 2:1 ratio. R_f = 0.30 (hexane/EtOAc = 0/100 v/v)

5-[(4-Methoxyphenyl)amino]-7-methylnon-8-ene-1,2,3,4-tetraol (**2**). (a mixture of major and minor isomers in a ratio of 2:1): IR (neat) 3400 (m), 2930 (m), 1653 (s), 1539 (s), 1456 (s), 1231 (s), 1180 (s), 1038 (s), 916 (s), 829 (s), 735 (s), 667 (s) cm^{-1}; ^{1}H-NMR ($CDCl_3$, major-isomer) δ 0.98 (d, *J* = 6.6 Hz, 3 H), 1.48–1.57 (br d, *J* = 13.8 Hz, 2 H), 1.74 (ddd, *J* = 13.8, 10.4, 2.9 Hz, 1 H), 2.27–2.29 (br d, *J* = 7.7 Hz, 1 H), 3.61–3.69 (m, 2 H), 3.74 (s, 3 H), 3.76–3.90 (m, 3 H), 4.82 (d, *J* = 17.1 Hz, 1 H), 4.92 (dd, *J* = 10.2, 1.7 Hz, 1 H), 5.60 (ddd, *J* = 17.1, 10.2, 7.7 Hz, 1 H), 6.78 (d, *J* = 10.6 Hz, 2 H), 6.79 (d, *J* = 10.6 Hz, 2 H); ^{13}C-NMR ($CDCl_3$, major-isomer) δ 21.7, 35.2, 37.0, 55.9, 57.5, 63.6, 73.0, 73.4, 73.9, 114.4, 115.0, 117.4, 143.9, 153.9, 162.5; ^{1}H-NMR ($CDCl_3$, minor-isomer) δ 0.96 (d, *J* = 6.8 Hz, 3 H), 1.48–1.57 (br d, *J* = 13.8 Hz, 2 H), 1.74 (ddd, *J* = 13.8, 10.4, 2.9 Hz, 1 H), 2.27–2.29 (br d, *J* = 7.7 Hz, 1 H), 3.61–3.69 (m, 2 H), 3.75 (s, 3 H), 3.76–3.90 (m, 3 H), 4.82 (d, *J* = 17.1 Hz, 1 H), 4.91 (d, *J* = 11.2 Hz, 1 H), 5.60 (ddd, *J* = 17.1, 10.2, 7.7 Hz, 1 H), 6.78 (d, *J* = 10.6 Hz, 2 H), 6.79 (d, *J* = 10.6 Hz, 2 H); ^{13}C-NMR ($CDCl_3$, minor-isomer) δ 21.7, 34.9, 36.6, 55.9, 57.5, 63.6, 73.2, 73.7, 73.9, 114.4, 115.1, 117.8, 144.2, 153.9, 162.5; HRMS, calcd for $C_{17}H_{27}NO_5$: 325.1889. Found *m*/*z* (relative intensity): 326.1940 (M^++1, 20), 325.1873 (M^+, 100).

(2R, 3S, 4R, 6S, 8S)-6-(4-Methoxyphenylamino)-8-methyl-9-decen-1,2,3,4-tetraol (**3**). (a mixture of major and minor isomers in a ratio of 1:1): IR (KBr) 3285 (s), 2937 (m), 2924 (m), 1514 (s), 1412 (m), 1240 (s), 1074 (s), 1040 (s), 822 (w), 640 (w) cm^{-1}; ^{1}H-NMR ($CDCl_3$, major-isomer) δ 1.00 (d, *J* = 6.8 Hz, 3 H), 1.44–1.62 (m, 4 H), 2.04–2.11 (ddd, *J* = 5.1, 9.3, 12.7, 1 H), 2.26 (br d, *J* = 7.5 Hz, 1 H), 3.51–3.83 (m, 5 H), 3.74 (s, 3 H), 4.16–4.20 (m, 1 H), 4.89 (d, *J* = 18.0 Hz, 1 H), 4.92 (d, *J* = 10.5 Hz, 1 H), 5.63 (ddd, *J* = 17.1, 10.2, 7.8 Hz, 1 H), 6.73 (d, *J* = 9.0 Hz, 2 H), 6.77 (d, *J* = 9.0 Hz, 2 H); ^{13}C-NMR ($CDCl_3$, major-isomer) δ 20.8, 35.2, 37.9, 42.5, 51.7, 55.9, 64.0, 68.9, 72.9, 74.7, 113.7, 115.0, 116.7, 140.8, 144.1, 153.3; ^{1}H-NMR ($CDCl_3$, minor-isomer) δ 0.93 (d, *J* = 6.8 Hz, 3 H), 1.44–1.62 (m, 4 H), 2.04–2.11 (ddd, *J* = 5.1, 9.3, 12.7, 1 H), 2.26 (br d, *J* = 7.5 Hz, 1 H), 3.51–3.83 (m, 5 H), 3.76 (s, 3 H), 4.16–4.20 (m, 1 H), 4.88 (d, *J* = 17.3 Hz, 1 H), 4.97 (d, *J* = 9.3 Hz, 1 H), 5.63 (ddd, *J* = 17.1, 10.2, 7.8 Hz, 1 H), 6.72 (d, *J* = 10.7 Hz, 2 H), 6.79 (d, *J* = 9.0 Hz, 2 H); ^{13}C-NMR ($CDCl_3$, minor-isomer) δ 20.6, 35.0, 37.6, 42.9, 51.7, 55.5, 64.2, 68.9, 73.0, 74.3, 113.5, 115.0, 116.7, 140.8, 144.3, 153.3; HRMS, calcd for $C_{18}H_{29}NO_5$: 339.2046. Found *m*/*z* (relative intensity): 340.2072 (M^++1, 20), 339.2037 (M^+, 100).

References

1. Kodato, S.; Nakagawa, M.; Nakayama, K.; Hino, T. Synthesis of cerebroside B_{1b} with antiulcerogenic activity I. Synthesis of ceramides with optically active α-hydroxypalmitic acids. *Tetrahedron* **1989**, *45*, 7247–7262.
2. Mukai, R.; Horino, Y.; Tanaka, S.; Tamaru, Y.; Kimura, M. Pd(0)-catalyzed amphiphilic activation of bis-allyl alcohol and ether. *J. Am. Chem. Soc.* **2004**, *126*, 11139–11138.

3. Kimura, M.; Shimizu, M.; Tanaka, S.; Tamaru, Y. Pd-catalyzed nucleophilic allylic alkylation of aliphatic aldehydes by the use of allyl alcohols. *Tetrahedron* **2005**, *61*, 3709–3718.
4. Yamaguchi, Y.; Hasimoto, M.; Tohyama, K.; Kimura, M. Nucleophilic allylation of *N*,*O*-acetals with allylic alcohols promoted by Pd/Et_3B and Pd/Et_2Zn systems. *Tetrahedron Lett*. **2011**, *52*, 913–915.
